# Supplementary material for: CD16/PD-L1 bi-specific aptamer for cancer immunotherapy through recruiting NK cells and acting as immunocheckpoint blockade
Source: Mol Ther Nucleic Acids. 2022 Jan 19;27:998–1009. doi: 10.1016/j.omtn.2022.01.010 (PMC8844804; doi:10.1016/j.omtn.2022.01.010)
Supplement: Document S1. Figures S1–S17 [file mmc1.pdf]

OMTN, Volume *27*

Supplemental information

CD16/PD-L1 bi-specific aptamer for cancer immunotherapy through re-cruiting NK cells and acting as immunocheckpoint blockade

Aixian Zheng, Yanlin Du, Yiru Wang, Youshi Zheng, Zhaoyu Ning, Ming Wu, Cuilin Zhang, Da Zhang, Jingfeng Liu, and Xiaolong Liu

Table S1. The sequences of aptamers used in this study

Name	Detailed sequence
CD16-apt	5'-(P)-GACCTGCCCACTGCGGGGGTCTATACGTGAGGAAGAAGTGGGCAGGTCCAGACGGGCTTTTTTTTTTTTTTTTTTTTTTTTTTTTTTTTTGCCCGTCTG-3'
PD-L1-apt	5'-(P)-GACCTGCCCACTTTTTTTTTTTTTTTTTTTTTTTTAGTGGGCAGGTCCAGACGGGCCACATCAACTCATTGATAGACAATGCGTCCACTGCCCGTCTG-3'
CPmut-bi-apt	5'-(P)-GACCTGCCCACTGCGGGGGTCTATACGTGAGGAAGAAGTGGGCAGGTCCAGACGGGCCACATCAACTCATT***C***ATA***C***ACAAT***C***C***C***TCCACTGCCCGTCTG-3'
CmutP-bi-apt	5'-(P)-GACCTGCCCACT***C***CGG***C***GGT***G***TATAC***C***TGAG***C***AAGAAGTGGGCAGGTCCAGACGGGCCACATCAACTCATTGATAGACAATGCGTCCACTGCCCGTCTG-3'
CP-bi-apt	5'-(P)-GACCTGCCCACTGCGGGGGTCTATACGTGAGGAAGAAGTGGGCAGGTCCAGACGGGCCACATCAACTCATTGATAGACAATGCGTCCACTGCCCGTCTG-3'

The letters with straight underline and wavy underline represent the sequences of CD16 aptamer and PD-L1 aptamer, respectively. CD16-apt and PD-L1-apt were constructed by only replacing the bases of counter aptamer in the loop of the CP-bi-apt with T bases. CmutP-bi-apt and CP$_{mut}$-bi-apt are the aptamers with some mutations, which were marked in bold italics. All of them are used as control.

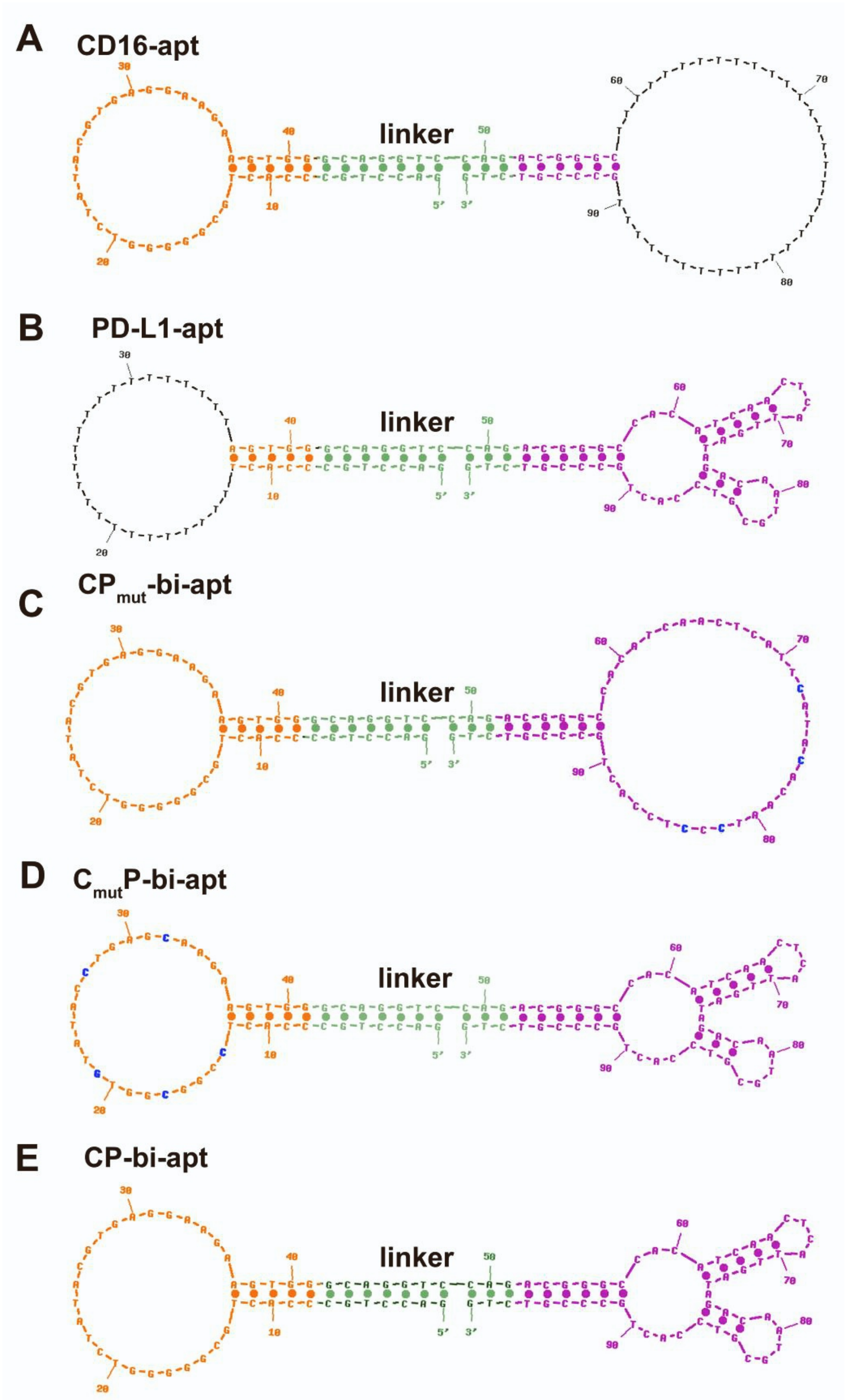

Figure S1. The predicted structures of the aptamers used in this study. The letters in green represent the sequences of linker.

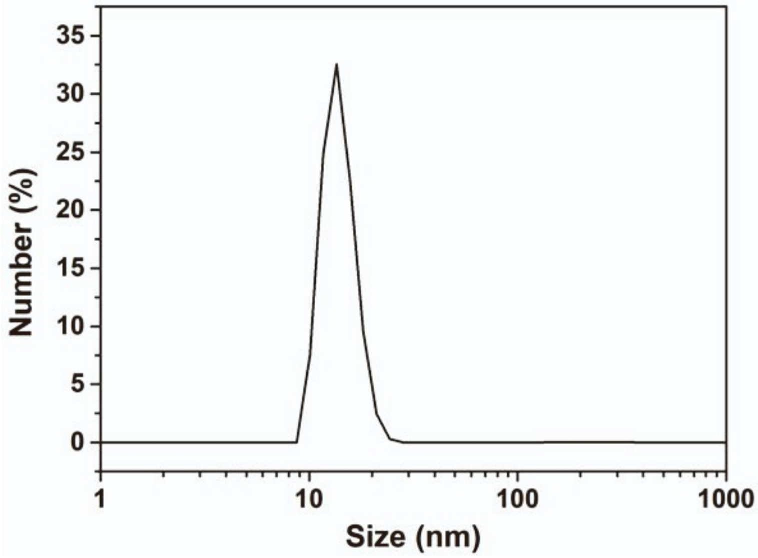

Figure S2. The hydrodynamic size of CP-bi-apt measured by Dynamic Light Scattering (DLS).

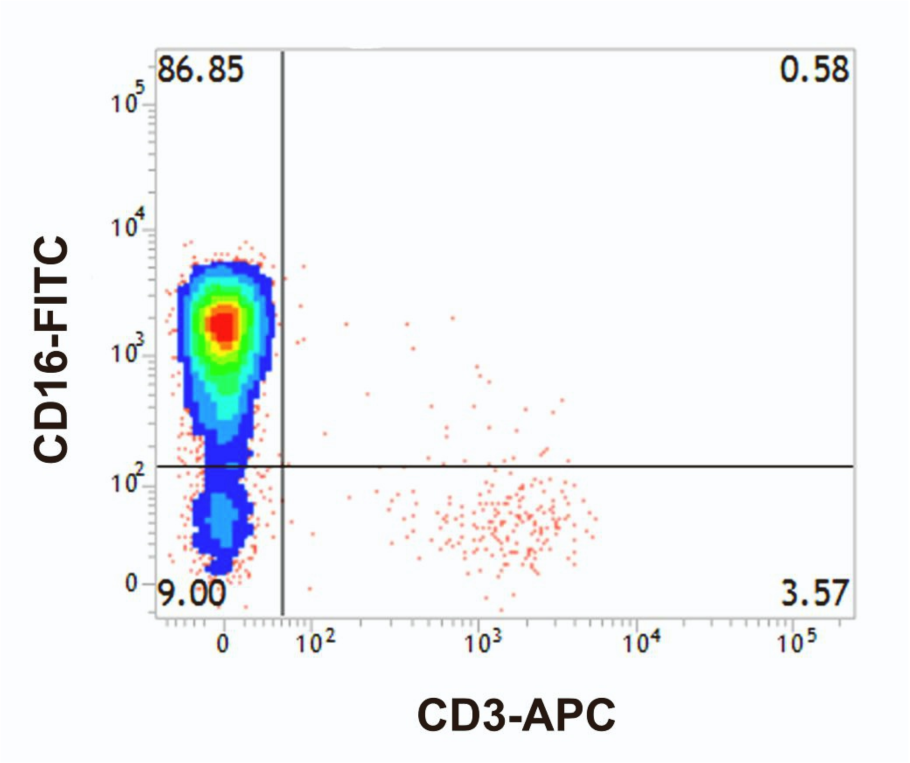

Figure S3. The expression of CD16 in human NK cells. NK cells were stained with anti-CD3-APC and anti-CD16-FITC before determining by flow cytometry.

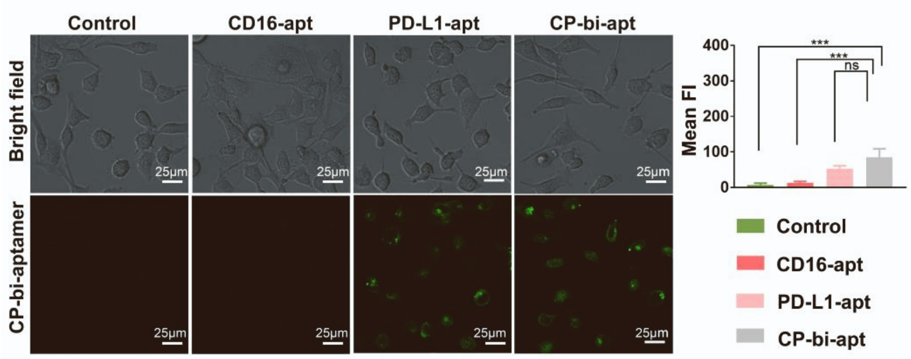

Figure S4. The binding ability of CP-bi-apt to QSG-7701 cells. QSG-7701 cells were incubated with 200 nM Evagreen-labeled different aptamers (CD16-apt, PD-L1-apt and CP-bi-apt) for 30 min. The fluorescence images were detected by confocal microscope (488 nm).

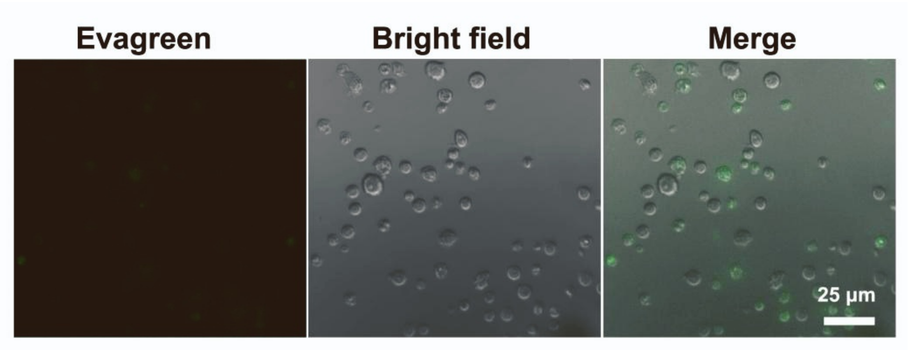

Figure S5. Confocal fluorescence images of murine NK cells incubated with Evagreen labeled CP-bi-apt (200 nM).

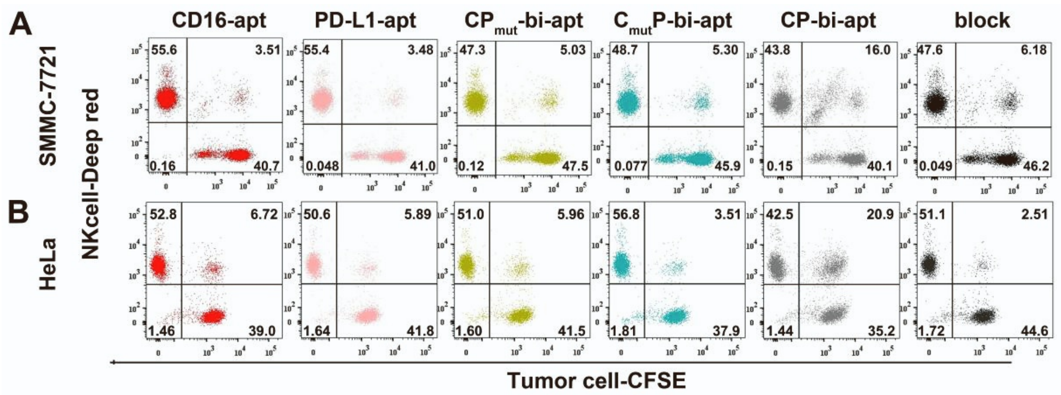

Figure S6. Flow cytometric analysis of CFSE-labeled (A) SMMC-7721 cells or (B) HeLa cells after co-incubating with Cell-Tracker Deep Red-labeled NK cells at 37℃ for 60 min. NK cells were pretreated with different aptamers (PD-L1-apt, CD16-apt, C_{mut}P-bi-apt, CP_{mut}-bi-apt or CP-bi-apt) at 37℃ for 30 min.

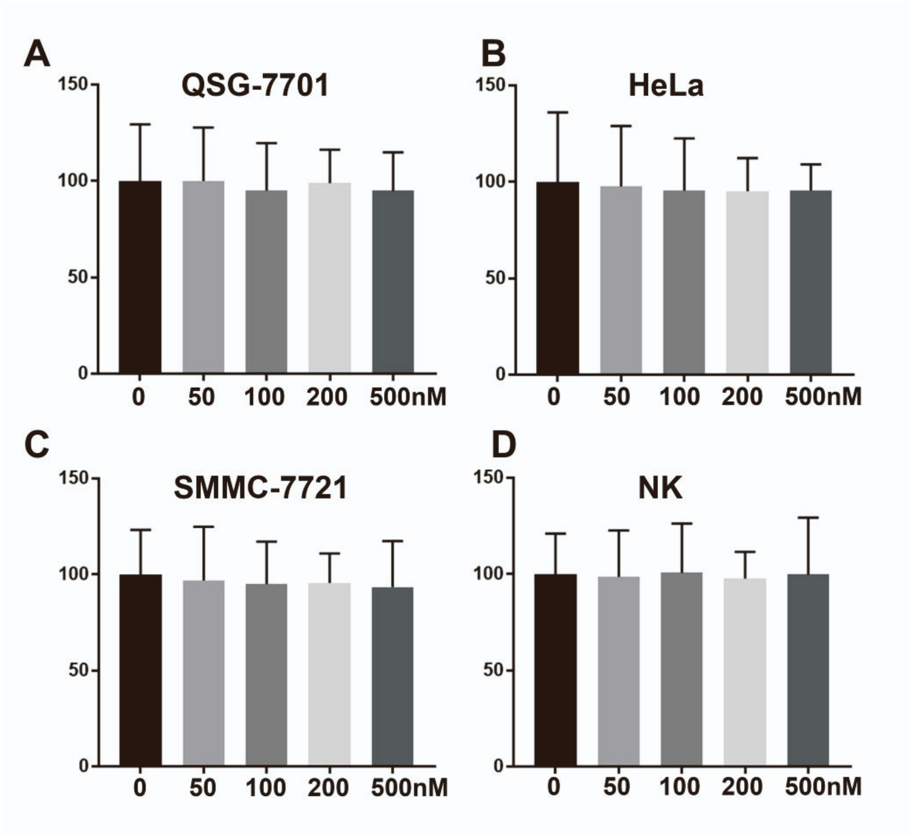

Figure S7. The in vitro toxicity of CP-bi-apt in different cells analyzed by CCK8 assay. Cell viability of (A) QSG-7701, (B) HeLa, (C) SMMC-7721 and (D) NK cells after co-incubation with different concentrations of CP-bi-apt (0, 50, 100, 200, 500 nM) for 24 h.

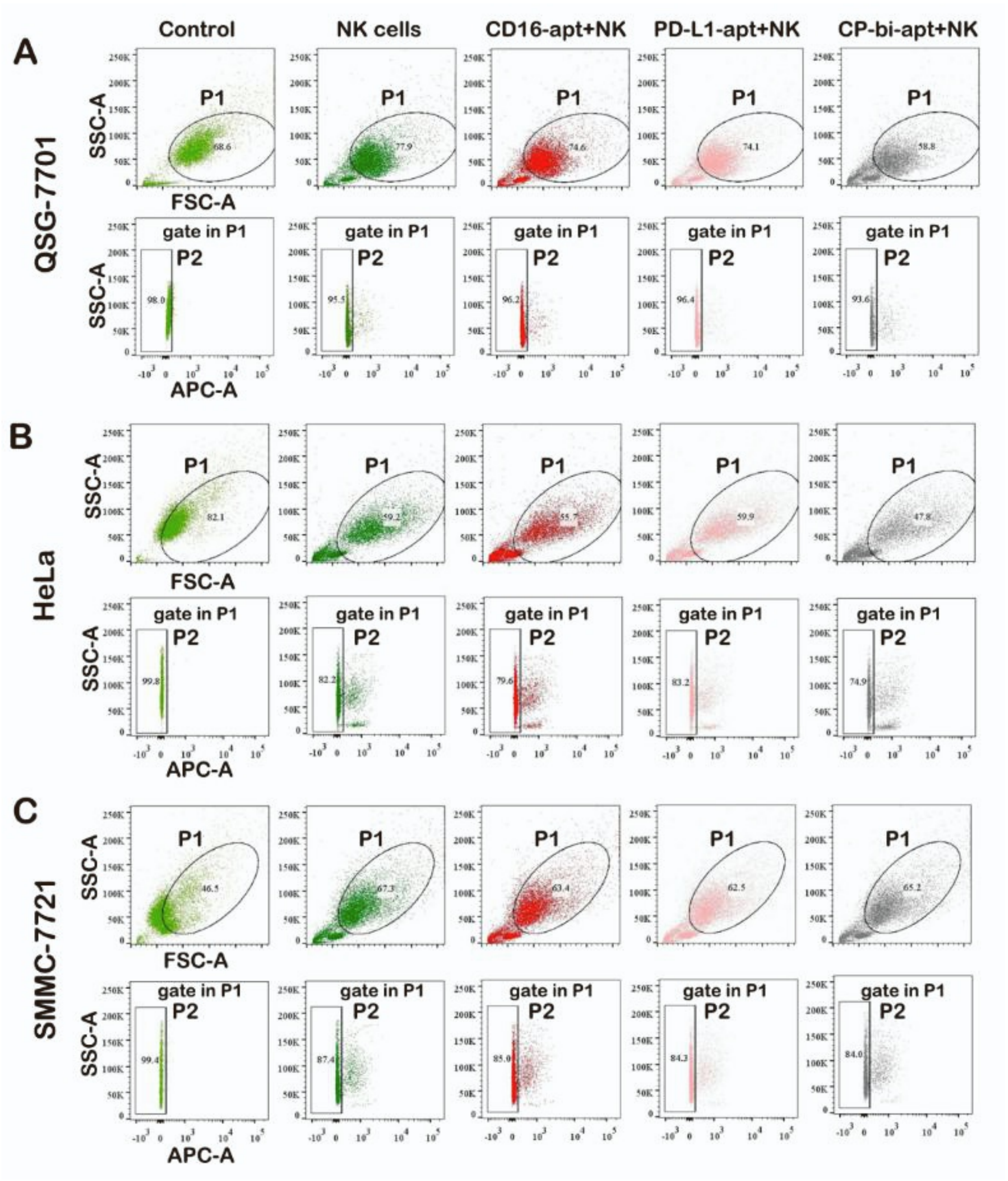

Figure S8. The gating strategy for flow cytometry analysis of (A) QSG-7701, (B) HeLa and (C) SMMC-7721 cells in the presence of NK cells and different aptamers (PD-L1-apt, CD16-apt or CP-bi-apt).

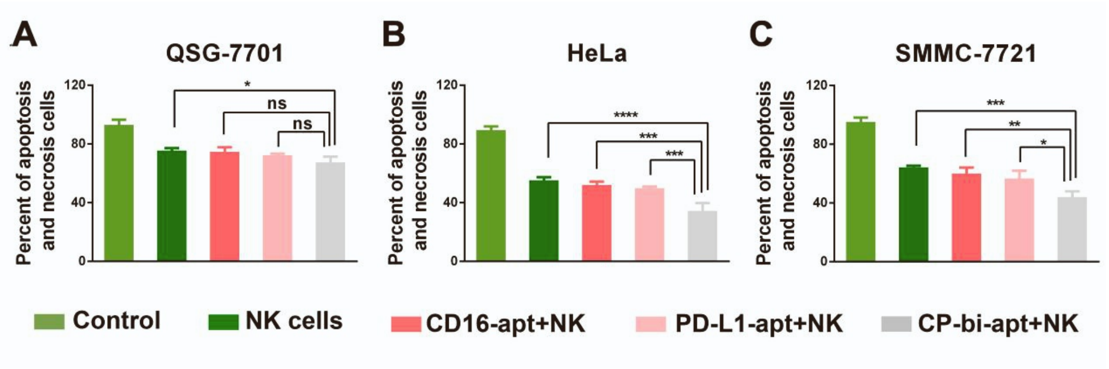

Figure S9. Flow cytometry analysis for evaluating the killing effect. Percentage of apoptosis and necrosis of (A) QSG-7701, (B) HeLa and (C) SMMC-7721cells after incubation with NK cells and different aptamers. The data represents the average result of three independent experiments.

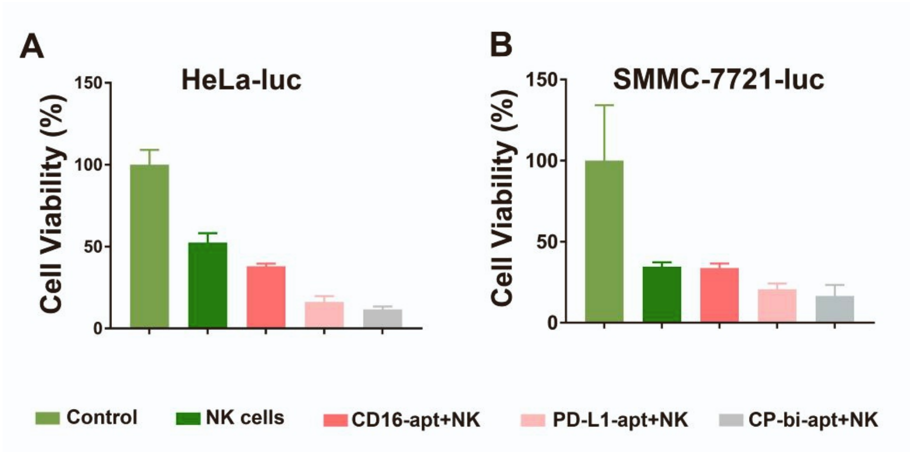

Figure S10. The luciferase based lysis assay for evaluating the killing effect. Cell viability of (A) HeLa-luc and (B) SMMC-7721-luc after co-incubation with NK cells and different kinds of aptamers (PD-L1-apt, CD16-apt or CP-bi-apt) for 3h.

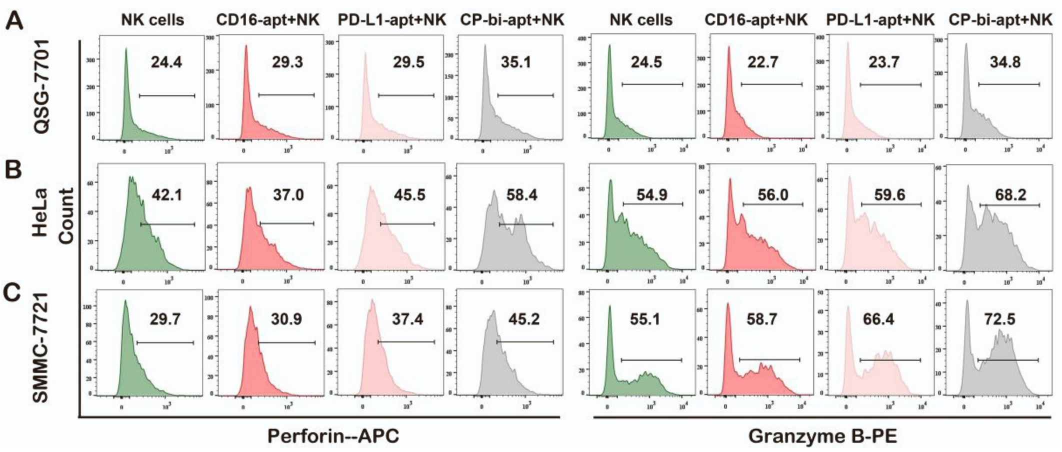

Figure S11. Flow cytometric analysis of intracellular Granzyme B and perforin of NK cells after co-incubating with different aptamers and (D) QSG-7701, (E) HeLa or (F) SMMC-7721 cells. The above experiments are all gated on CD56(+) cells by using FITC-conjugated anti-CD56 antibody.

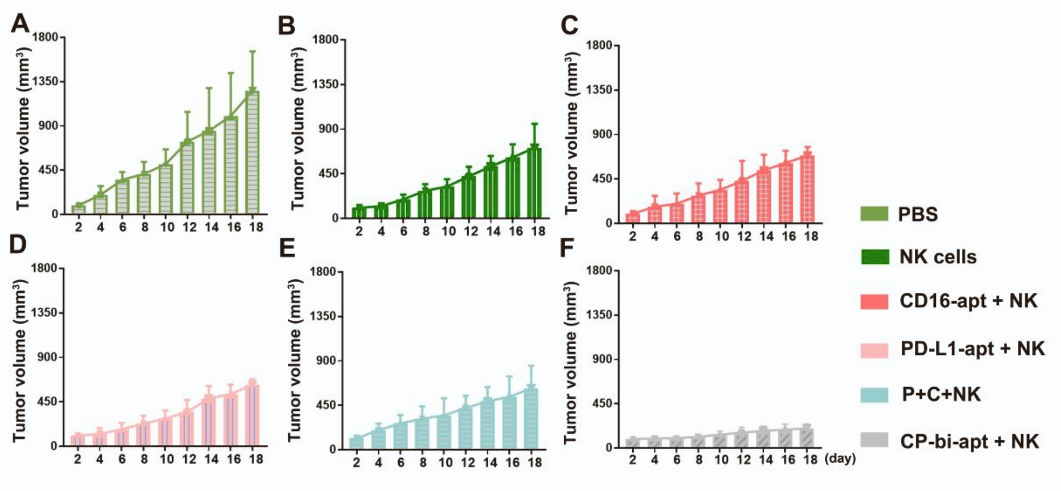

Figure S12. The individual growth curves of the tumors after various treatments (PBS, free NK cells, CD16-apt + NK cells, PD-L1-apt + NK cells, CD16-apt + PD-L1-apt +NK cells, CP-bi-apt + NK cells, n=5).

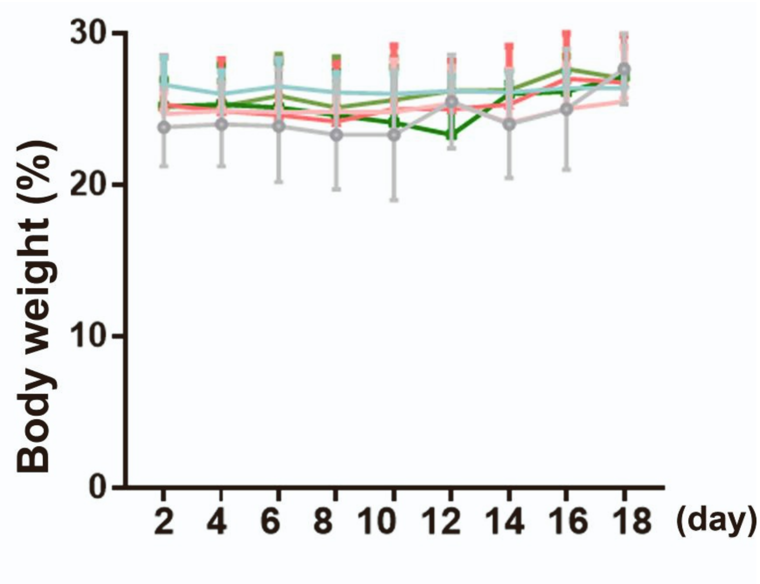

Figure S13. The body weights of the mice after various treatments (PBS, free NK cells, CD16-apt + NK cells, PD-L1-apt + NK cells, CD16-apt + PD-L1-apt +NK cells, CP-bi-apt + NK cells, n=5).

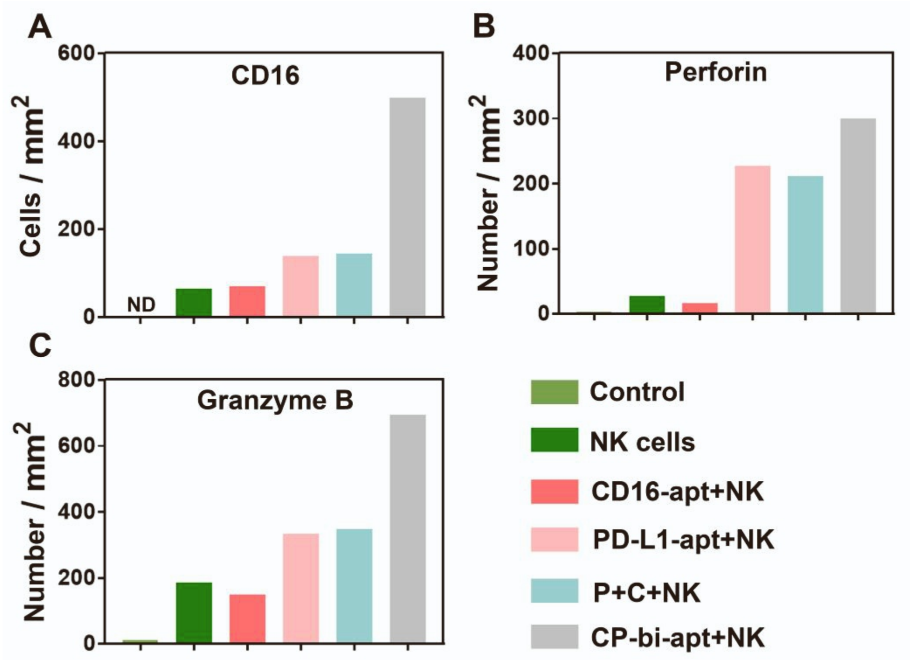

Figure S14. The statistical results of (A) CD16, (B) perforin and (C) Granzyme B immunofluorescence staining shown in Figure 6.

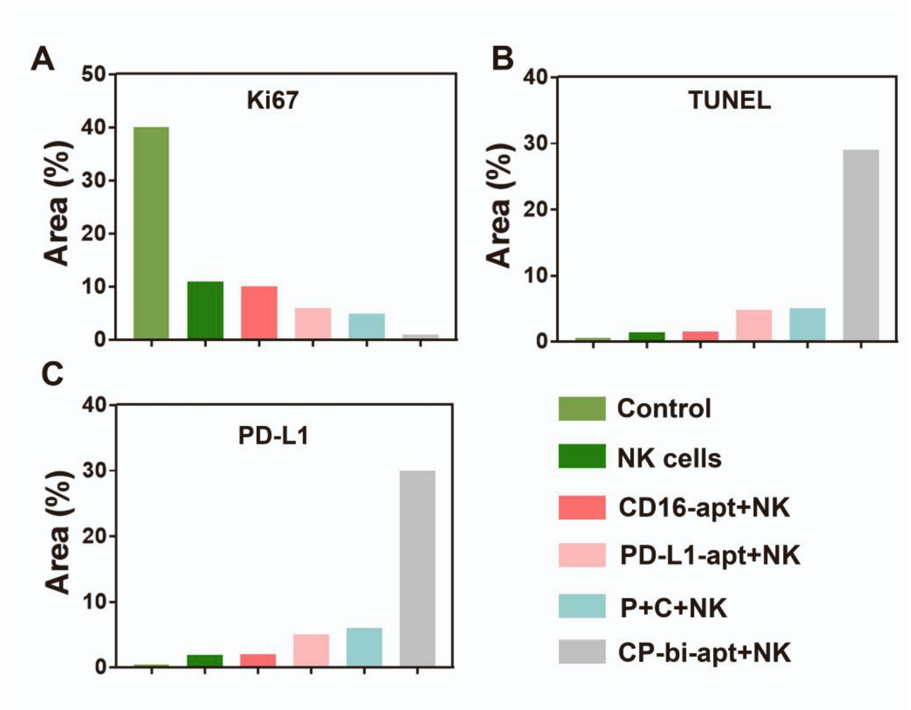

Figure S15. The statistical results of (A) Ki67, (B) TUNEL and (C) PD-L1 staining shown in Figure 7.

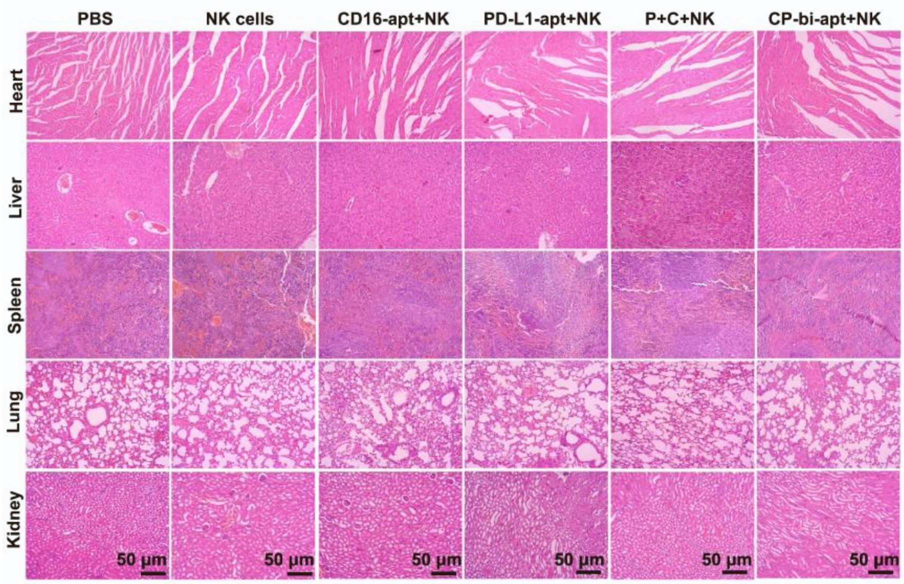

Figure S16. Histological images of the major organs (heart, liver, spleen, lung, and kidney) of mice with indicated treatments after the staining with H&E. Scale bar: 50 μm.

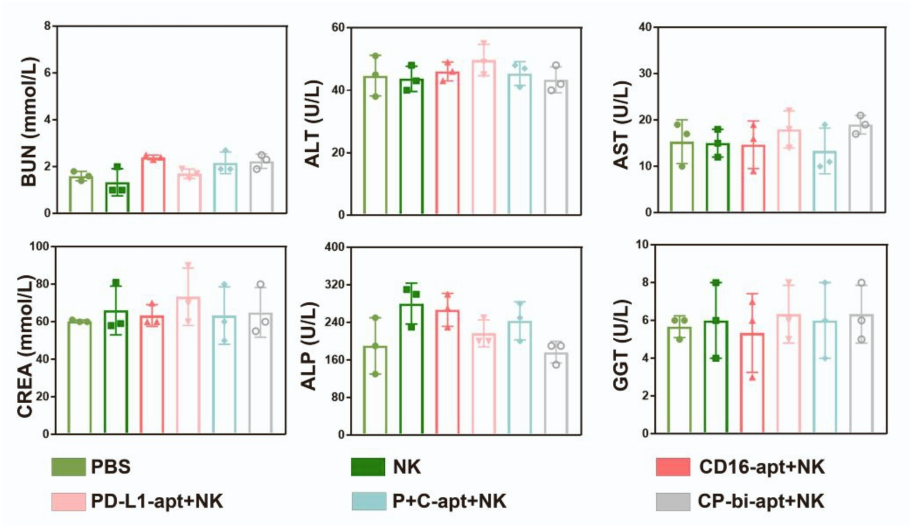

Figure S17. The analysis of serum biochemical indexes of the SMMC-7721 tumor-bearing mice with indicated treatments at three days after last injection. n = 3.
