# Supplementary material for: CD16/PD-L1 bi-specific aptamer for cancer immunotherapy through recruiting NK cells and acting as immunocheckpoint blockade
Source: Mol Ther Nucleic Acids. 2022 Jan 19;27:998–1009. doi: 10.1016/j.omtn.2022.01.010 (PMC8844804; doi:10.1016/j.omtn.2022.01.010)
Supplement: Document S2. Article plus supplemental information [file mmc2.pdf]

Molecular Therapy
Nucleic Acids
Original Article

CD16/PD-L1 bi-specific aptamer for cancer immunotherapy through recruiting NK cells and acting as immunocheckpoint blockade

Aixian Zheng,[1,6] Yanlin Du,[1,3,4,6] Yiru Wang,[1,5,6] Youshi Zheng,[1] Zhaoyu Ning,[1,5] Ming Wu,[1] Cuilin Zhang,[1] Da Zhang,[1] Jingfeng Liu,[1,2] and Xiaolong Liu[1,2,3]

[1]The United Innovation of Mengchao Hepatobiliary Technology Key Laboratory of Fujian Province, Mengchao Hepatobiliary Hospital of Fujian Medical University, Fuzhou 350025, P.R. China; [2]The Hepatobiliary Medical Center of Fujian Province, Fujian Cancer Hospital & Fujian Medical University Cancer Hospital, Fuzhou 350014, P.R. China; [3]Fujian Institute of Research on the Structure of Matter, Chinese Academy of Sciences, Fuzhou 350002, P.R. China; [4]College of Life Science, Fujian Agriculture and Forestry University, Fuzhou 350002, P.R. China; [5]College of Biological Science and Engineering, Fuzhou University, Fuzhou 350116, P.R. China

It is well established that natural killer (NK) cells can be used as an alternative candidate of T cells for adoptive cell therapy (ACT) due to its high killing capacity, off-the-shelf utility, and low toxicity. Though NK cells provide rapid and potent immune effects, they still suffer from insufficient infiltration and tumor immunosuppression environment, which result in unsatisfactory therapeutic efficiency. Herein, a highly stable CD16/PD-L1 bi-specific aptamer (defined as CP-bi-apt) with high affinity and selectivity was introduced to overcome these obstacles. This CP-bi-apt can mediate a significant antitumor immunity by recruiting CD16-positive NK cells to directly contact with PD-L1 high-expressed tumor cells. In addition, the induced up-regulation of PD-L1 on tumor cells can inevitably occur as an adaptive response to most of the immunotherapeutic strategies. The prepared CP-bi-apt can be further used as an immune checkpoint inhibitor to specifically bind to PD-L1, thus reducing the negative impact of PD-L1 over-expression on the therapeutic efficacy. Furthermore, this CP-bi-apt-based immunotherapy is simple, highly efficient, and has low side effects, showing a promising potential for clinical translation.

INTRODUCTION

Adoptive cell therapy (ACT) has become one of the most flexible and potent cancer treatments that can elicit partial and even complete regression of various malignancies.[1] Despite the robust antitumor capability of T-cell-based ACT, it still encounters several limitations, such as the requirement of strict autologous histocompatibility leukocyte antigen (HLA) matching as well as the risk of neurologic toxicity, cytokine storm syndrome, and graft-versus-host disease (GVHD).[2–5] In contrast to T cells, natural killer (NK) cells do not depend on antigen-specific receptors or somatic rearrangement but recognize target cells through germ-line encoded receptors.[6,7] This inherent property of NK cells enables them to rapidly respond to specific signals without prior sensitization or clonal expansion, thus playing an important role in innate and adaptive immunity.[8,9] Owing to the advantages of high killing capacity, low cost, off-the-shelf utility, and safety to the body, NK cells are used as an attractive candidate for T cells in ACT.[10,11] However, the insufficient infiltration and the low abundance of NK cells in solid tumors can only lead to limited therapeutic success.[12,13] To address this problem, great efforts have been implemented to endow NK cells with the functions of tumor homing and infiltration. To this aim, NK cells are usually modified by genetic engineering or chemical methods, which are relatively tedious and hard to reproduce and may also cause new safety concerns.

Aptamers are unnatural single-stranded oligonucleotides that can specifically bind to their targets, including small molecules, proteins, viruses, and even cells by self-folding into special three-dimensional structures.[14,15] Explicitly, aptamers and aptamer-based nanoparticles have attracted wide attention in immunotherapies due to their low immunogenicity, flexible modification, and low toxicity.[16–23] Bi-specific aptamer (bi-apt) is formed by two monomeric aptamers, which can specifically bind to two kinds of targets with high affinity.[24,25] Recently, a variety of bi-apts have been constructed for antitumor immunotherapies.[26–28] They can be used to recruit T cells, NK cells, or other immune cells, thereby enhancing the antitumor immune responses.[29–31] However, the stability of bi-apts *in vivo* is still a worrying problem, which may limit their further clinical applications.

It is well known that immune checkpoints play an important role in the maintenance of immune homeostasis under normal physiological conditions. However, immune checkpoints also show their negative

Received 27 April 2021; accepted 17 January 2022;
https://doi.org/10.1016/j.omtn.2022.01.010.

[6]These authors contributed equally

Correspondence: Xiaolong Liu, PhD, The United Innovation of Mengchao Hepatobiliary Technology Key Laboratory of Fujian Province, Mengchao Hepatobiliary Hospital of Fujian Medical University, Fuzhou 350025, P.R. China.
E-mail: xiaoloong.liu@gmail.com
Correspondence: Jingfeng Liu, MD, PhD, The Hepatobiliary Medical Center of Fujian Province, Fujian Cancer Hospital & Fujian Medical University Cancer Hospital, Fuzhou 350014, P.R. China.
E-mail: drjingfeng@126.com

This is an open access article under the CC BY license (http://creativecommons.org/licenses/by/4.0/).

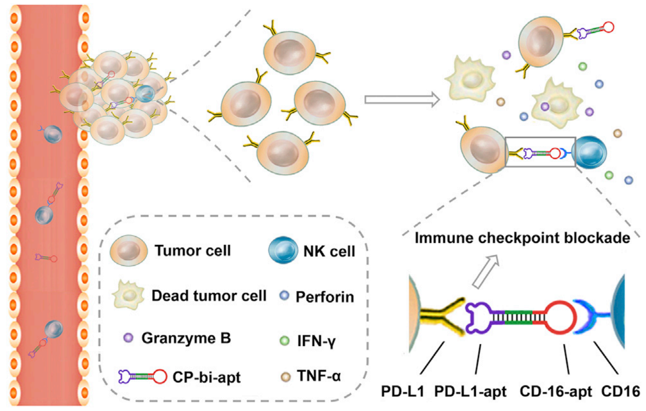

Figure 1. Schematic illustration of CP-bi-apt for enhancing antitumor immunotherapy

Adoptive NK cells can induce robust immune response with the engagement of CP-bi-apt. Moreover, CP-bi-apt also can enhance the antitumor effect through PD-L1 immune checkpoint blockade.

tradeoffs, which can be adopted by tumor cells to escape the clearance by immune cells. Similar to T cells, NK cells also express immune checkpoints, including PD-1, which can decrease the antitumor effect of NK cells when combined with their corresponding ligands.[32] Fortunately, the blockade of immune checkpoint can restore the function of immune cells and thus provoke robust antitumor immune response.[33,34] It has been reported that CD16 is over-expressed on NK cells,[35] while PD-L1 is generally expressed on tumors.[36] Thus, utilizing PD-L1 immune checkpoint as the binding site to recruit NK cells to tumors may provide a great opportunity to address the aforementioned problems of NK-cell-based immunotherapies and thus significantly improve the antitumor effect.

Herein, a novel CD16/PD-L1 dual-targeting aptamer (CP-bi-apt) was established for mediating NK-cell-based antitumor immunity. This aptamer can be used for the recruitment of NK cells to directly contact with PD-L1 high-expressed tumor cells, thus enhancing the antitumor immune response (Figure 1). To palliate the degradation especially *in vivo*, CP-bi-apt was endowed with a highly stable and completely closed structure with the help of T4 DNA ligase.[30,37] During the treatment, NK cells will secrete cytokines that directly induce cancer cell death or promote antitumor immunity. It is worth mentioning that interferon (IFN)-γ is essential for antitumor immunity, which has been reported to induce the up-regulation of PD-L1 on tumor cells.[38,39] Fortunately, the injected CP-bi-apt also can be used as an immune checkpoint inhibitor to block PD-L1 and thus reduce the influence of PD-L1 over-expression on the therapeutic efficacy. It is hoped that the CP-bi-apt-based immunotherapy will pave the way for further clinical translation due to its remarkable antitumor effect.

RESULTS

Preparation and characterization of CP-bi-apt

In order to obtain a better therapeutic effect, a novel bi-apt (CP-bi-apt) was designed and then synthesized by Shanghai Sangon Biotechnology (Shanghai, China), which is mainly composed of CD16 aptamer[24] (aptCD16) and PD-L1 aptamer[40] (aptPD-L1). To address the steric hindrance between them, oligonucleotides with 10 bp were used as a linker to connect these two monomeric aptamers into a semi-closed dumbbell structure. To improve the stability of CP-bi-apt, its 5' end and 3' end were modified with phosphate and hydroxyl, respectively. It can form a highly stable and completely closed structure with the help of T4 DNA ligase. The nucleic acids only containing aptCD16 or aptPD-L1 (CD16-apt and PD-L1-apt) were used as control. In order to form highly stable and completely closed structures similar to CP-bi-apt, thereby reducing the influence of nucleic acid structure themselves, CD16-apt and PD-L1-apt were constructed only by replacing the bases of counter-aptamer in the loop of CP-bi-apt with T bases. Meanwhile, the aptamers with some mutations (C_{mut}P-bi-apt and CP_{mut}-bi-apt) have also been used as control to support the specific binding of CP-bi-apt to CD16 or PD-L1. All the sequences of the aptamers used in this study were shown in Table S1. Meanwhile, the predicted structures of them were shown in Figure S1. Due to the relatively small size of this aptamer, dynamic light scattering (DLS) was used for characterization. It can be found that the hydrated size of CP-bi-apt was about 13.5 nm (Figure S2), which matched well with the predicted structure of CP-bi-apt.
The completely closed structure of the aptamer was characterized by electrophoresis analysis. As shown in Figure 2A, the bands of the aptamer treated or untreated with T4 DNA ligase were almost the same in 2% agarose gel, indicating that the sequence of nucleic acid has not changed, while the bands of them were obviously different in denaturing polyacrylamide gel, indicating the change of the nucleic acid structure after treating with T4 DNA ligase (Figure 2B). As shown in Figure 2C, the untreated aptamer was completely degraded after reacting with nuclease I/III for 6 h, while the aptamer treated with T4 DNA ligase can be stable for at least 24 h. These results indicated that the prepared CP-bi-apt has formed a completely closed structure, which is more resistant to nuclease degradation. Moreover, when dissolved in 50% fetal bovine serum (FBS), the band of the untreated aptamer was almost invisible after 24 h, while the aptamer treated with T4 DNA ligase still showed strong signal (Figure 2D). All these results demonstrated that CP-bi-apt has been successfully prepared, the stability of which was obviously improved.

The expression of CD16 in NK cells and PD-L1 in cancer cells

The CP-bi-apt was constructed based on the specific targeting of CD-16 and PD-L1. To ensure that the designed CP-bi-apt can play the designed role in antitumor immunity, the expression of PD-L1 in human cancer cells and CD16 in human NK cells was analyzed, respectively. As shown in Figure 2E, PD-L1 was highly expressed in cancer cell lines (HeLa and SMMC-7721), while it was lowly expressed in normal cells (QSG-7701 cells). Besides, the proportion of CD16-positive NK cells in the maturated NK cells was more than 86% (Figure S3), which was analyzed by flow cytometry.

The specificity of CP-bi-apt and the ability to recruit NK cells

We then evaluated whether CP-bi-apt could specifically recognize its corresponding target cells. To this end, HeLa, SMMC-7721,

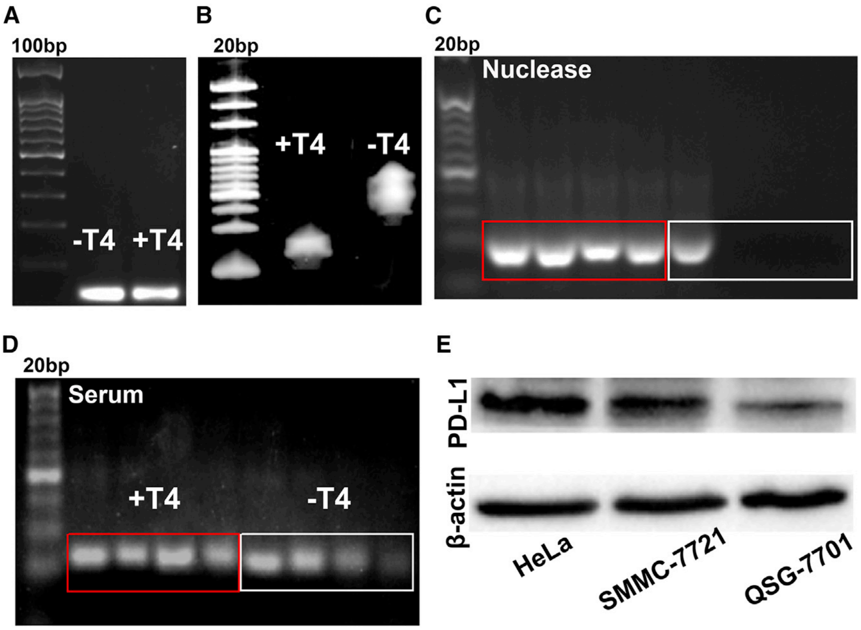

Figure 2. Characterization and stability analysis of CP-bi-apt

(A and B) The analysis of CP-bi-apt with/without the treatment of T4 DNA ligase under (A) 2% agarose gel electrophoresis and (B) 7 M-urea-containing polyacrylamide gel electrophoresis. (C and D) The stability of CP-bi-apt with or without the treatment of T4 DNA ligase after incubation in (C) nuclease I/III or (D) 50% FBS at different time points (0 h, 6 h, 12 h, and 24 h) is shown. (E) The expression of PD-L1 in HeLa, SMMC-7721, and QSG-7701 cells.

QSG-7701, and NK cells were incubated with Evagreen-labeled different aptamers (CD16-apt, PD-L1-apt, C_{mut}P-bi-apt, CP_{mut}-bi-apt, or CP-bi-apt), respectively. Then, the fluorescence images were observed with confocal microscope. After the incubation of CP-bi-apt, C_{mut}P-bi-apt, or PD-L1-apt, noticeable fluorescence signals were observed in PD-L1 high-expressed SMMC-7721 cells (Figure 3A) and HeLa cells (Figure 3B), while the fluorescent signal was much weaker in PD-L1 low-expressed QSG-7701 cells (Figure S4). In addition to these, obvious fluorescence signals were obtained in NK cells with the treatment of CP-bi-apt, CP_{mut}-bi-apt, or CD16-apt (Figure 3C), which all contain CD-16 aptamer. These results confirmed that the prepared CP-bi-apt could specifically bind to cancer cells and NK cells through aptPD-L1 and aptCD16, respectively. Of interest is that the fluorescence signals of tumor cells and NK cells were significantly reduced when they were pre-treated with anti-PD-L1 or anti-CD16 antibodies. All these results can prove that CP-bi-apt has the ability to specifically bind to PD-L1 expressed on tumor cells and CD16 expressed on NK cells. We further assessed whether the CP-bi-apt could bind to murine NK cells, which were selected from the spleens of nude mice by using a Mouse NK Cell Isolation Kit. The isolated murine NK cells were incubated with 200 nM Evagreen-labeled CP-bi-apt and then resuspended in PBS for confocal fluorescence imaging. As shown in Figure S5, CP-bi-apt could not bind well to murine NK cells according to the weak fluorescence signal, indicating the specificity of CP-bi-apt to human NK cells.

The high affinity of CP-bi-apt toward NK cells and PD-L1 high-expressed tumor cells has been demonstrated. However, its ability to tether the two types of cells together remains unknown. For tracking purposes, tumor cells were labeled with carboxyfluorescein succinimidyl ester (CFSE) (green) and NK cells were labeled with Cell-Tracker Deep Red (red) and then they were co-incubated in the presence of different aptamers (PD-L1-apt, CD16-apt, C_{mut}P-bi-apt, CP_{mut}-bi-apt, or CP-bi-apt). After co-incubation, flow cytometry analysis was then used to determine the engagement between NK cells and tumor cells. As shown in Figure 3D, the percentage of double-positive cell populations in CP-bi-apt-treated SMMC-7721 cell and NK cell mixtures was 21.1%, which was significantly higher than that in other aptamers-treated cell mixtures. It can be found that the double-positive cells showed one or more populations in different groups. This may be because cancer cells can be connected with different numbers of NK cells, resulting in different cell populations. When SMMC-7721 cells were pre-treated with anti-PD-L1 antibody and then incubated with NK cells and CP-bi-apt, the percentage of double-positive cell populations decreased to 6.36%. Similar results also can be obtained in HeLa cell and NK cell mixtures (Figure 3E). These results indicated that CP-bi-apt can specifically bind to PD-L1 highly expressed tumor cells and CD16-positive NK cells and then tether them together. We then investigated whether the pre-binding of CP-bi-apt to CD16-positive NK cells would mask the effect of PD-L1 aptamer targeting. Briefly, Cell-Tracker-Deep-Red-labeled NK cells were pre-treated with different aptamers (PD-L1-apt, CD16-apt, CmutP-bi-apt, CPmut-bi-apt, or CP-bi-apt) for 30 min and then co-incubated with CFSE-labeled cancer cells. After co-incubation, the cell mixture was subjected to flow cytometric analysis. We can find that the percentage of double-positive cell populations in cancer cell and CP-bi-apt pre-treated NK cell mixture was still significantly higher than that in other aptamers-treated cell mixtures (Figure S6). These data suggested that CP-bi-apt can assist to direct NK cells to tumor cells, even if CP-bi-apt was pre-bound with NK cells.

CP-bi-apt enhances the killing effect of NK cells

No toxicity or low toxicity is the premise of biomedical applications. Therefore, the cytotoxicity of CP-bi-apt in different cells was firstly evaluated by Cell Counting Kit 8 (CCK8) assay. As shown in Figure S7, with the increase of CP-bi-apt concentration, there were no significant differences in cell viabilities among QSG-7701, HeLa,

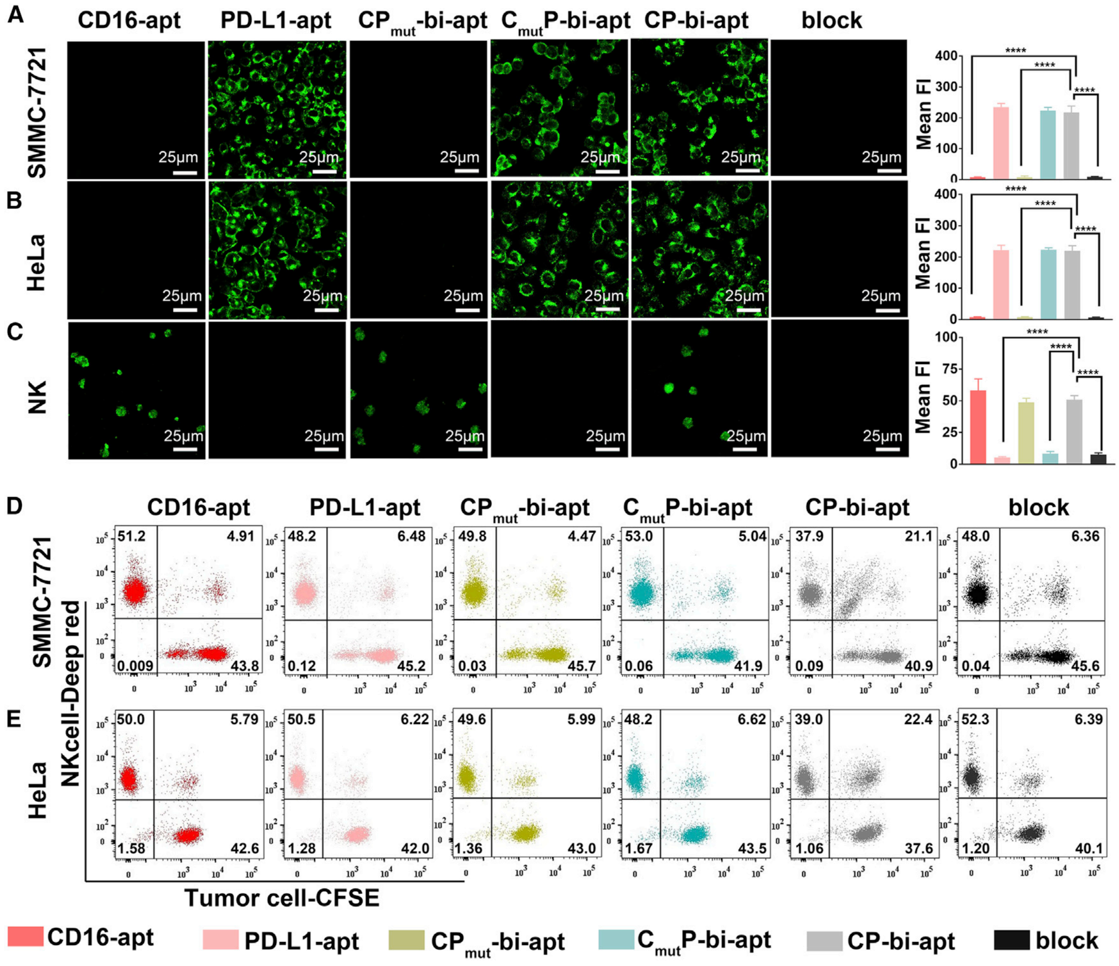

Figure 3. Specific recognition of tumor cells and NK cells by CP-bi-apt

(A–C) Confocal fluorescence images of (A) SMMC-7721, (B) HeLa, and (C) NK cells incubated with Evagreen-labeled aptamers as indicated (200 nM). In block group, the cells were pre-treated with anti-PD-L1 antibody or anti-CD16 antibody and then incubated with CP-bi-apt. Data are presented as mean ± SEM (error bar); ****p<0.0001. (D and E) Flow cytometric analysis of CFSE-labeled (D) SMMC-7721 cells or (E) HeLa cells after co-incubating with Cell-Tracker-Deep-Red-labeled NK cells (E:T = 1:1) and different aptamers (PD-L1-apt, CD16-apt, C_{mut}P-bi-apt, CP_{mut}-bi-apt, or CP-bi-apt) is shown.

SMMC-7721, and NK cells, respectively. Even if the concentration of CP-bi-apt reached 500 nM, the survival rates of these cells were still more than 90%, showing that CP-bi-apt itself has low toxicity and does not cause obvious damage to various cells.

We further evaluated whether CP-bi-apt could enhance the killing effect of NK cells on cancer cells. For this purpose, the normal and tumor cells were co-incubated with the corresponding aptamers and NK cells (effector/target [E/T] = 1:1) for 3 h. After that, the suspension cells and adherent cells in the medium were collected and then stained with anti-CD16-APC and fluorescein isothiocyanate (FITC)-annexin V/propidium iodide (PI) for flow cytometry analysis. The gating strategy for this experiment was posted in Figure S8. As illustrated in Figure 4A, QSG-7701 cells showed 23.1% of apoptosis or necrosis after the treatment with NK cells. Since QSG-7701 cells also express a small amount of PD-L1, the percentage of apoptotic and necrotic cells increased slightly further after extra addition of CP-bi-apt, while, for HeLa cells, the population of apoptotic and necrotic cells was as high as 46.8% after the incubation of NK cells, and it further increased to 50.9% and 70.0% after extra addition of PD-L1-apt and CP-bi-apt, respectively (Figure 4B). The SMMC-7721 cells experienced a similar trend that the population of apoptotic and necrotic cells increased from 36.4% (in NK cells group) to 49.4% and 53.0% with the assistance of PD-L1-apt and CP-bi-apt, respectively (Figure 4C). The statistics data with three repeat experiments have also been provided in Figure S9, in which the killing effect of NK cells on tumor cells assisted by CP-bi-apt was statistically significant. These results confirmed that the killing ability of NK cells on tumor cells could be enhanced by blocking the PD-L1 immune checkpoint. Meanwhile, CP-bi-apt could further enhance the killing effect of NK cells on tumor cells by recruiting NK cells to directly contact with tumor cells. The luciferase-based lysis assay was further used

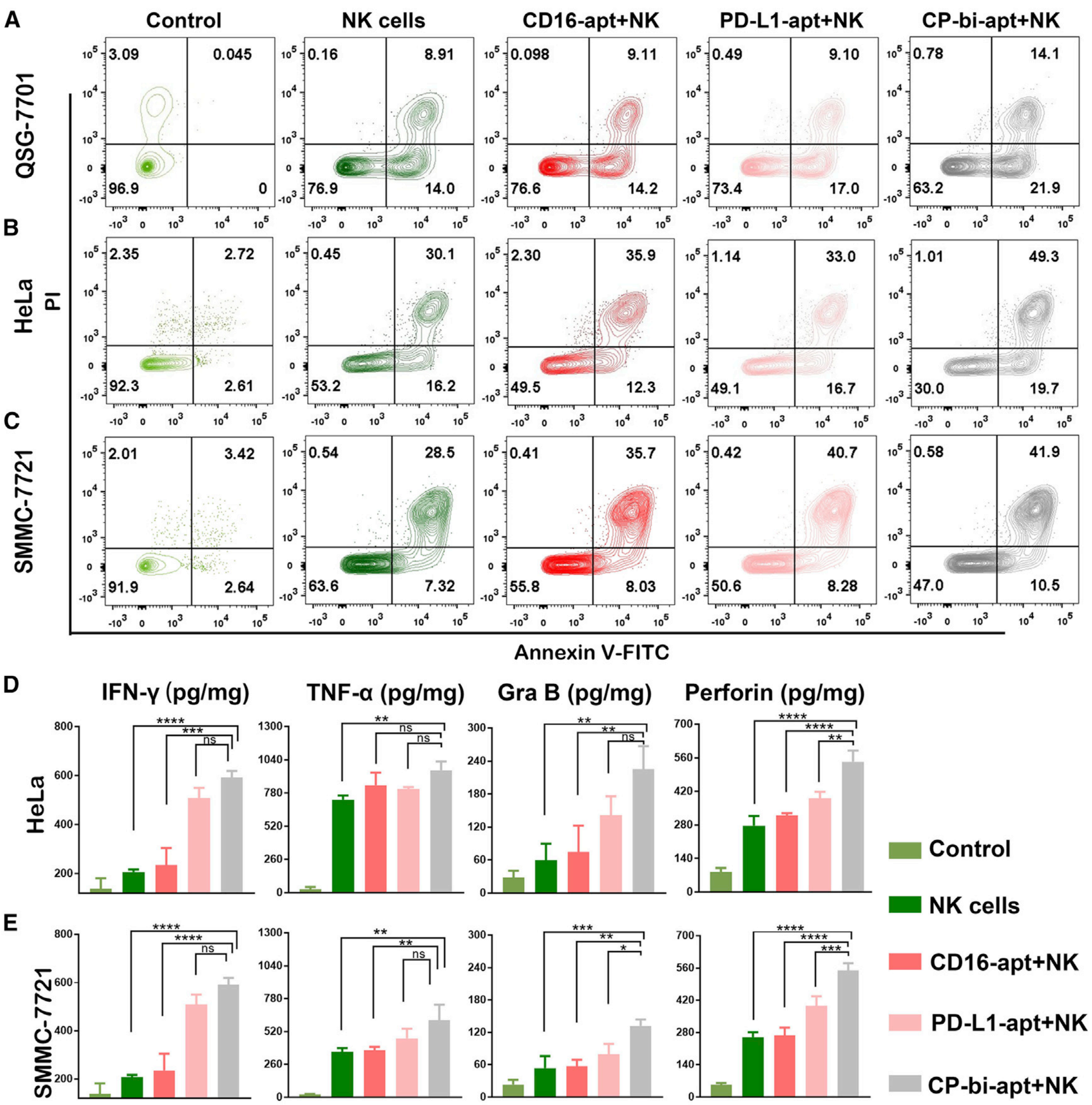

Figure 4. CP-bi-apt enhances the killing effect of NK cells

(A–C) Flow cytometry analysis of the cell activity of (A) QSG-7701, (B) HeLa, and (C) SMMC-7721 in the presence of NK cells and different aptamers. The above experiments were all gated on CD16 (–) cells by using antigen-presenting cell (APC)-conjugated anti-CD16 antibody. (D and E) The analysis of IFN-γ, TNF-α, granzyme B, and perforin in supernatants of (D) HeLa cells and (E) SMMC-7721 cells after indicated treatment is shown; n = 3.

to evaluate the killing effect of NK cells with the help of different aptamers. As shown in Figure S10, the viability of HeLa cells decreased to 53.7% after the incubation with NK cells, and it could further decrease to 11.6% after extra addition of CP-bi-apt. Similar results also can be obtained in SMMC-7721 cells, in which the cell viability further decreased from 34.6% to 16.5% after extra addition of CP-bi-apt. These results further proved that CP-bi-apt can enhance the killing effect of NK cells.

To further evaluate the killing effect of NK cells assisted by CP-bi-apt, cytokine secretion of IFN-γ, tumor necrosis factor alpha (TNF-α), granzyme B, and perforin was determined by ELISA assay. No matter whether HeLa cells or SMMC-7721 cells, when they were co-incubated with NK cells or different aptamers were added at the same time, the concentrations of the four cytokines in the medium increased significantly, especially in the presence of CP-bi-apt (Figures 4D and 4E). Meanwhile, intracellular cytokines (granzyme B and perforin) of NK cells were also analyzed by flow cytometry. As shown in Figure S11, the granzyme-B- and perforin-positive NK cells only slightly increased when co-incubated with QSG-7701 cells and CP-bi-apt, while, when co-incubated with HeLa cells and CP-bi-apt, the granzyme-B- and perforin-positive NK cells increased more

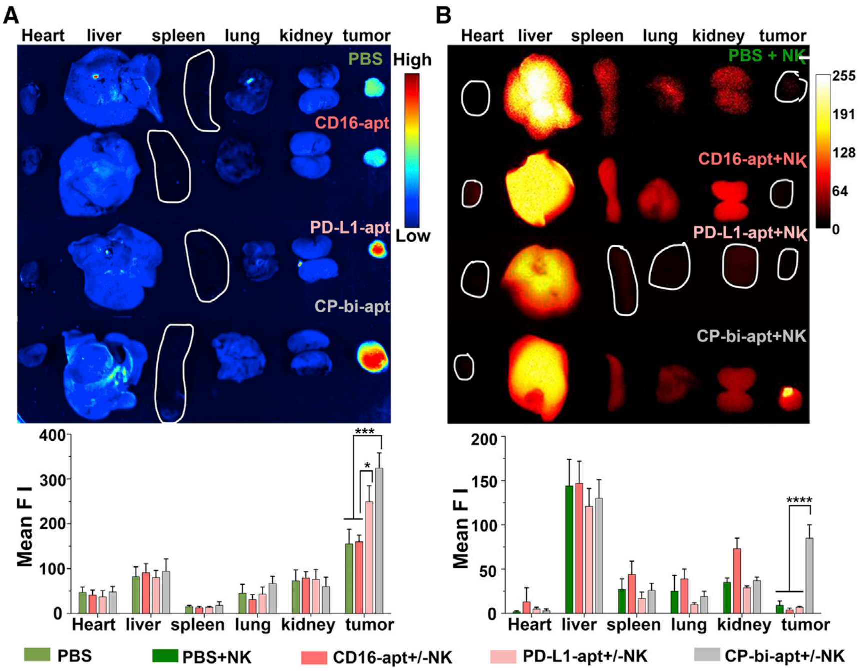

Figure 5. *In vivo* biodistribution of different aptamers and adoptive NK cells

(A) The fluorescence images of major organs and tumors isolated from tumor-bearing nude mice at 24 h after intravenous injection of PBS or Evagreen-labeled different aptamers (CD16-apt, PD-L1-apt, and CP-bi-apt; Ex: 488 nm); (B) *in vivo* biodistribution of ICG-NK cells after indicated treatments (Ex: 808 nm). The mean fluorescence intensity (Mean F I) was measured by Image(J). Data are presented as mean ± SEM; *$p<0.05$, ***$p<0.001$.*

significantly, reaching 68.2% and 58.4%, respectively. Similarly, the granzyme-B- and perforin-positive NK cells could increase to 72.5% and 45.2%, respectively, after co-incubation with SMMC-7721 cells and CP-bi-apt. These results were consistent with the killing effect analyzed by flow cytometry, further suggesting that CP-bi-apt can enhance the antitumor ability of NK cells.

In vivo biodistribution of CP-bi-apt and adoptive NK cells

We further investigated the *in vivo* biodistribution of CP-bi-apt in tumor-bearing nude mice. PBS, CD16-apt, and PD-L1-apt were used as control. For tracking purposes, all aptamers were labeled with Evagreen before injection. Then, they were injected intravenously into SMMC-7721 tumor-bearing mice until the tumor volume reached to a certain size. After 24 h, the major organs (heart, liver, spleen, lung, and kidney) as well as tumors were dissected to monitor the distribution of aptamers, which were imaged by Gel Doc XR imaging system. The fluorescence intensity of each tissue was quantified by ImageJ. As shown in Figure 5A, the fluorescence intensities of tumor tissues in CP-bi-apt- and PD-L1-apt-treated groups were significantly higher than that in CD16-apt- and PBS-treated groups. This phenomenon is due to the ability of both PD-L1-apt and CP-bi-apt to specifically bind to PD-L1, which is highly expressed on tumor cells.

We have demonstrated that CP-bi-apt could mediate the binding of NK cells to tumor cells at the cellular level. Afterward, we further investigated whether CP-bi-apt could promote the accumulation of NK cells in tumor sites. To confirm this, indocyanine green (ICG)-labeled NK cells and different aptamers were simultaneously injected into SMMC-7721 tumor-bearing mice. At 24 h post-injection, the tumor and major organs were excised for fluorescence imaging (Figure 5B). We can find that the fluorescence in tumor sites was very weak when only ICG-labeled NK cells were injected as well as when only CD16-apt or PD-L1-apt were injected at the same time. However, when ICG-labeled NK cells and CP-bi-apt were simultaneously injected, the fluorescence at the tumor site significantly increased, implying that more NK cells were recruited into the tumor tissue. This may be because CP-bi-apt can specifically bind to PD-L1 highly expressed tumor cells and CD16-positive NK cells, which may further assist to direct NK cells into the tumor site. Therefore, CP-bi-apt is expected to promote the accumulation and contacting of adoptive NK cells to tumors and thus enhance the antitumor effect.

In vivo antitumor effect of CP-bi-apt and adoptive NK cells

We then further evaluated whether the prepared CP-bi-apt could promote *in vivo* antitumor effect by the recruitment of NK cells to tumor site and then blocking PD-1/PD-L1 axis. Here, SMMC-7721 tumor-bearing nude mice were used, which were randomly divided into six groups for various treatments when the tumor volume reached 100 mm^3. As expected, the mice in treated groups showed a delayed tumor growth compared with PBS group (Figures 6A and S12). Furthermore, the group simultaneously treated with CP-bi-apt and NK cells showed the most dramatic tumor growth inhibition, while there was no significant difference in body weight change between the treated groups and the control group (Figure S13). To verify the significant therapeutic effect resulted from the activation of antitumor immunity, the pro-inflammatory cytokines of the treated mice were analyzed by ELISA. As shown in Figures 6B–6E, after the treatment with CP-bi-apt and NK cells simultaneously, the highest levels of TNF-α, IFN-γ, granzyme B, and perforin were observed. In addition, the tumor infiltration of NK cells is necessary for adaptive antitumor immunity. As shown in Figures 6F and S14A, the infiltration of NK cells (red) in tumor tissue was significantly enhanced with the help of CP-bi-apt, thus playing stronger antitumor response. The intracellular cytokine staining of NK cells has also been performed with CD56 as the marker for staining, which has also been reported to highly express on NK cells. As shown in Figures 6G, 6H, S14B, and S14C, the group simultaneously treated with CP-bi-apt and NK cells could produce more granzyme B and perforin, and only part of them could

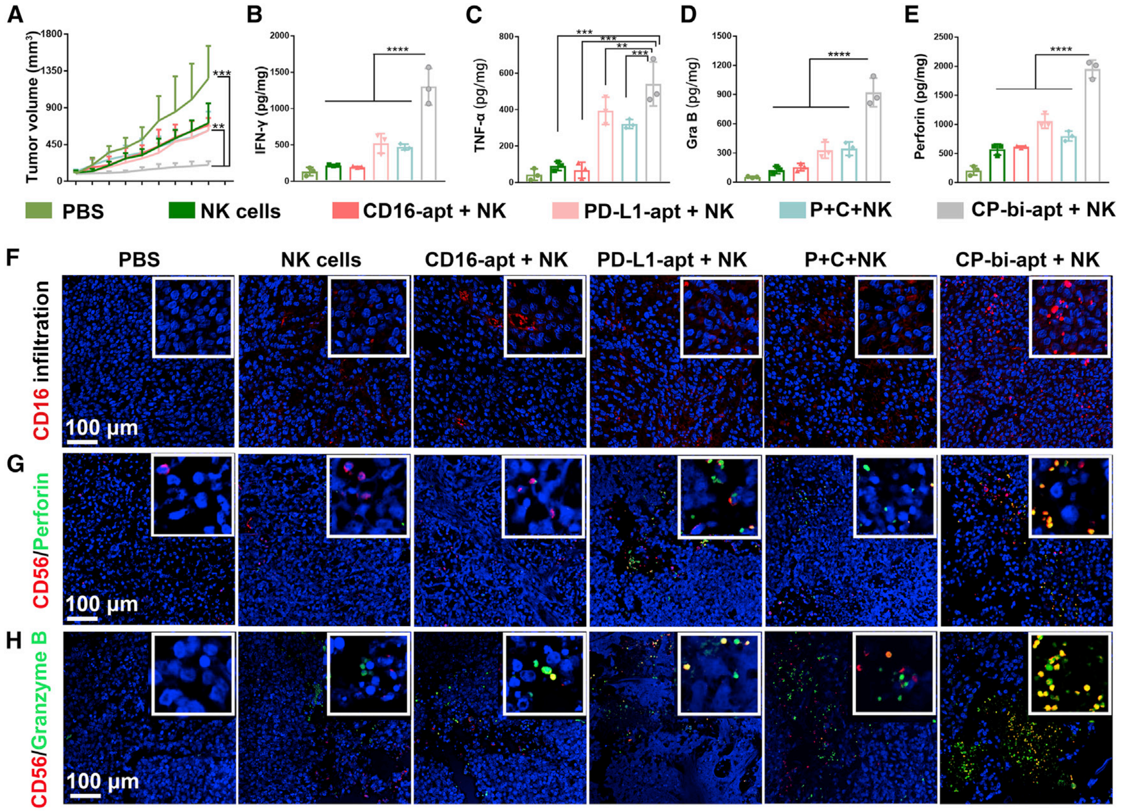

Figure 6. *In vivo* antitumor effect of different aptamers and adoptive NK cells

(A) The average growth curves of the tumors after various treatments (PBS, free NK cells, CD16-apt + NK cells, PD-L1-apt + NK cells, CD16-apt + PD-L1-apt + NK cells, and CP-bi-apt + NK cells; n = 5). (B–E) The ELISA analysis of (B) IFN-γ, (C) TNF-α, (D) granzyme B, and (E) perforin in tumors isolated from mice at day 3 after the last injection (n = 3). Data are presented as mean ± SEM; $^{**}p<0.01$, $^{***}p<0.001$, $^{****}p<0.0001$. (F) The representative CD16 immunofluorescence staining of the infiltrated NK cells (red) in tumor . (G and H) Perforin (G) and granzyme B (H) immunofluorescence staining of the NK cells in tumors, and CD56 was used as the marker. Scale bars: 100 μm. The insets are the corresponding enlarged image in each condition.

co-localize with NK cells. It is because granzyme B and perforin are secreted proteins, which would be secreted from NK cells. These results further indicated that CP-bi-apt could mediate significantly enhanced antitumor immunity of NK cells.

After treatment, the staining of H&E, Ki67, and terminal deoxynucleotidyl transferase 2'-deoxyuridine 5'-triphosphate nick end labeling (TUNEL) in tumor tissues was performed to further investigate the antitumor effect. As for H&E staining, much larger areas of necrosis and apoptosis were observed after treating with CP-bi-apt and NK cells, while the tumor cells retained the normal morphology in control group (Figure 7A). Meanwhile, the results of Ki67 staining showed that tumor cells in the control group had obvious proliferation phenomenon, indicated by the brown granules in the cell nucleus (Figures 7B and S15A). However, after the treatment of CP-bi-apt and NK cells simultaneously, the positive rate of Ki67 was much lower. In addition, the most apoptotic cells with green fluorescence were observed in this group (Figures 7C and S15B). These results further proved that CP-bi-apt could enhance the killing effect of adoptive NK cells on tumor cells *in vivo*.

Previous studies reported that the over-expression of PD-L1 on most tumor cells can be induced by IFN-γ signaling as an adaptive response to antitumor immunity, which in turn will affect the efficacy of immunotherapies.[38,41] We then further investigated the PD-L1 over-expression in tumors after different treatments by immunohistochemistry. Compared with other groups, more significant up-regulation of PD-L1 expression was observed after the treatment of CP-bi-apt and NK cells simultaneously (Figures 7D and S15C). These data are consistent with more secretion of IFN-γ in the indicated treatment. Since we have demonstrated that CP-bi-apt could specifically bind to PD-L1 that expressed on cancer cells to act as an immune checkpoint inhibitor, multiple injections of CP-bi-apt may not only promote the accumulation of NK cells in tumor sites but also could reduce the influence of PD-L1 up-regulation, thus improving the antitumor effect.

Biosafety of CP-bi-apt-based treatment *in vivo*

The mice were sacrificed at 3 days after last injection, and the major organs (heart, liver, spleen, lung, and kidney) were stained with H&E to evaluate the biosafety of CP-bi-apt-based treatment *in vivo*. After

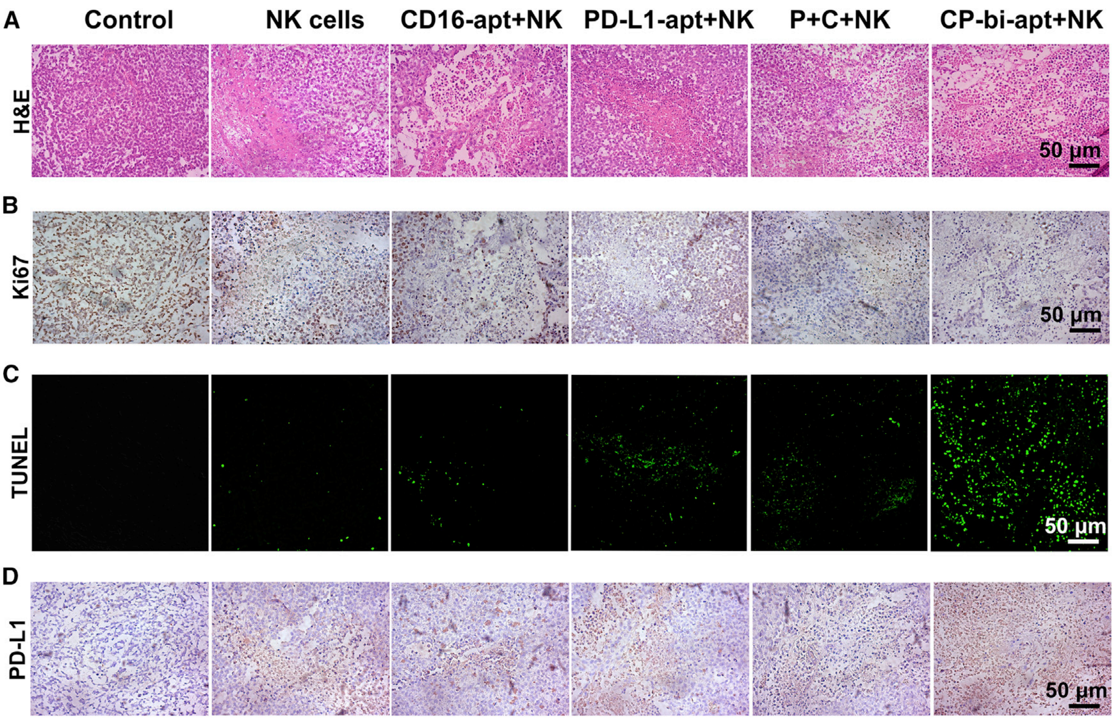

Figure 7. Histological analyses of antitumor efficacy

(A–C) Optical microscopic images of tumor slices stained by (A) H&E and (B) Ki67 and (C) confocal images of tumor slices after the immunofluorescence staining of TUNEL (green; Ex: 488 nm). (D) The PD-L1 staining of tumor slices at 3 days after last injection is shown. Scale bars: 50 μm.

different treatments, there were no significant morphological changes of the major organs compared with the PBS-treated mice (Figure S16). To further assess the biosafety, serum from the mice in each group was obtained for the analysis of serum biochemical indicators, containing alkaline phosphatase (ALP), alanine aminotransferase (ALT), aspartate aminotransferase (AST), blood urea nitrogen (BUN), creatinine (CREA), and gamma-glutamyltransferase (GGT). As shown in Figure S17, all these indicators of liver and kidney functions were within the normal range. All these results revealed the outstanding biocompatibility and biosafety of CP-bi-apt *in vivo*.

DISCUSSION

In summary, a novel CD16/PD-L1 bi-specific aptamer named CP-bi-apt was developed for cancer immunotherapy that integrates the functions of immune checkpoint blockade and the recruitment of NK cells to tumor site without any additional procedures. The prepared CP-bi-apt can form a completely closed structure with the help of T4 DNA ligase, which can significantly enhance its stability. This aptamer can mediate a significant antitumor immunity by recruiting CD16-positive NK cells to directly contact with PD-L1 high-expressed tumor cells. It is worth mentioning that the expression of PD-L1 on tumor cells can be induced as an adaptive response to antitumor immunity, but the injected CP-bi-apt also can be used as an immune checkpoint inhibitor to block up-regulated PD-L1, and thus, the function of NK cells can be restored, which promotes robust antitumor immune response. It can be found that the tumor growth of tumor-bearing mice was significantly inhibited, and no obvious toxicity and side effects were observed after the injection of NK cells and CP-bi-apt simultaneously. However, the therapeutic effect of this approach is significantly related to the expression of PD-L1 on tumor cells. For some tumor cells, the expression of PD-L1 may be not so obviously different from that on normal cells and then will have limited therapeutic efficacy. But for most tumors with high PD-L1 expression, the therapeutic effect would be significantly enhanced by this approach. It has been reported that PD-L1 can be also expressed on NK cells. Meanwhile, anti-PD-L1 antibody can enhance the function of PD-L1-positive NK cells against PD-L1-negative tumors in both mouse and human systems. In this way, CP-bi-apt may also bind to PD-L1 expressed on NK cells and thus enhance the therapeutic effect on PD-L1-negative tumors.[42,43] Thus, this work is expected to provide a simple and highly efficient bi-apt for immunotherapy, showing a promising prospect in clinical applications.

MATERIALS AND METHODS

Preparation of CP-bi-apt

CP-bi-apt was composed of aptCD16 and aptPD-L1, and the oligonucleotides with 10 bp were used as a linker to connect them. To improve the stability of CP-bi-apt, its 5’ end and 3’ end were modified with phosphate and hydroxyl, respectively. It can form a highly stable and completely closed structure with the help of T4 DNA ligase. The nucleic acids only containing aptCD16 or aptPD-L1 (CD16-apt and

PD-L1-apt) were used as control. All of them were synthesized by Shanghai Sangon Biotechnology (Shanghai, China). The predicted structures were shown in Figure S1. Moreover, the semi-closed aptamers (10 μM) were added to the solution containing T4 DNA ligase (Thermo Fisher Scientific, USA) and reacted overnight at 16°C to form a completely closed structure. Then, the products were stored at −20°C for subsequent experiments.

Gel electrophoresis

The structures of the aptamers with or without the treatment of T4 DNA ligase were evaluated by 2% agarose gel electrophoresis and denaturing 10% polyacrylamide gel electrophoresis (containing 7 M urea), respectively. Briefly, the agarose gel was run in 1× Tris-acetate-EDTA (TAE) buffer at 120 V for 30 min. Then, the image was visualized by using Gel Doc XR imaging system (Bio-Rad, Hercules, CA). Polyacrylamide gel was run in 1× TAE buffer at 100 V for 45 min. Then, the image was visualized by using Gel Doc XR imaging system after staining with SYBR GOLD (Thermo Fisher Scientific). As for stability assay, 8 μL of 50% FBS (Thermo Fisher Scientific) and 2 μL of CP-bi-apt (10 μM) were incubated at 37°C for different times (0 h, 6 h, 12 h, and 24 h), and then the integrity of aptamers was analyzed with 2% agarose gel electrophoresis. Besides, to verify that CP-bi-apt can avert the degradation by nucleases, nuclease I and III mixtures (Thermo Fisher Scientific) were added to CP-bi-apt (2 μL, 10 μM), and the subsequent process was consistent as above.

Cell culture

The human hepatocellular carcinoma cell line (SMMC-7721), human cervical cancer cell line (HeLa), and human normal hepatic cell line (QSG-7701) were maintained as a monolayer culture in DMEM medium (Thermo Fisher Scientific) supplemented with 10% FBS (PAN Biotech, Germany) and 1% streptomycin/penicillin (Thermo Fisher Scientific) that were incubated at 37°C in a humidified atmosphere (5% CO_2). In addition, human NK cells were selected from human peripheral blood mononuclear cells (PBMCs) by a density-gradient technique (GE Healthcare Bio-Sciences ABSE, Uppsala, Sweden) and were matured in serum-free KBM 581 medium (Thermo Fisher Scientific) containing interleukin-2 (IL-2) (Kingsley Pharmaceutical, China) and K562 feeder cells. Moreover, the ethical approval for the usage of human samples was granted by the Ethics Committee of Mengchao Hepatobiliary Hospital of Fujian Medical University.

Western blot

Proteins were extracted from different kinds of cells by using radioimmunoprecipitation assay (RIPA) buffer (Beyotime Biotechnology, China) supplemented with 1× halt protease inhibitor cocktail (MedChemExpress, USA). After centrifuging for 10 min at 4°C (12,000 × *g*), the supernatant was collected and quantified by a bicinchoninic acid (BCA) (TransGen Biotech, China) assay. The primary antibody against human protein PD-L1 (Abcam, Cambridge, UK) was used at a 1:1,000 dilution, and the secondary antibody was used at a 1:5,000 dilution (Abcam, Cambridge, UK). After a series of basic western blot procedures, the signal of PD-L1 expression was captured by exposing to Gel Doc XR imaging system. Finally, signal intensity was measured with ImageJ.

The specificity of CP-bi-apt

Commensurable QSG-7701, SMMC-7721, and HeLa cells (2×10^5) were seeded in confocal dishes for 12 h and then incubated with equimolar (200 nM) Evagreen-labeled (Biotium, China) aptamer (CD16-apt, PD-L1-apt, CP_{mut}-bi-apt, $C_{mut}P$-bi-apt, or CP-bi-apt) at 37°C for 30 min. The cells without adding aptamer were used as control. Subsequently, confocal microscope (Zeiss LSM780, USA) was used to examine the binding ability after removing the unbound aptamer by washing with PBS (GE Life Sciences, China). Moreover, NK cells (1×10^6) were incubated with equimolar (200 nM) Evagreen-labeled aptamer (CD16-apt, PD-L1-apt, CP_{mut}-bi-apt, $C_{mut}P$-bi-apt, or CP-bi-apt) at 37°C for 30 min. Then, cell mixture was centrifuged at 800 × *g* to remove the unbound aptamer. After washing with PBS, the NK cells were resuspended in 500 μL PBS and then placed on confocal dishes for fluorescence imaging. Finally, signal intensity was obtained by using the supporting imaging processing software.

We then further investigated the specificity of CP-bi-apt toward CD16 and PD-L1. Here, SMMC-7721/HeLa cells (2×10^5) were pre-treated with 1 μg/mL anti-PD-L1 antibody (Thermo Fisher Scientific) and NK cells (1×10^6) were pre-treated with 1 μg/mL anti-CD16 antibody (Thermo Fisher Scientific) at 37°C for 1 h, respectively. After co-incubation with 200 nM Evagreen-labeled CP-bi-apt for an additional 30 min (at 37°C), the cells were washed with PBS to remove the unbound aptamer and then recorded by confocal microscope. Finally, the fluorescence intensity was obtained by using the supporting imaging processing software.

Recruitment of NK cells

We further analyzed the recruitment of NK cells by flow cytometry.[23] Briefly, CFSE-labeled tumor cells (SMMC-7721 and HeLa cells; 2×10^5) were incubated with Cell-Tracker Deep Red (Thermo Fisher Scientific)-labeled NK cells at the ratio of 1:1 in the presence of equimolar (200 nM) different aptamers (CD16-apt, PD-L1-apt, CP_{mut}-bi-apt, $C_{mut}P$-bi-apt, or CP-bi-apt) at 37°C for 1 h. After that, the percentage of double-positive cell populations in tumor cell and NK cell mixtures were quantified by flow cytometry. To verify the specificity of CP-bi-apt to PD-L1, CFSE-labeled tumor cells were first blocked with anti-PD-L1 antibody and then incubated with CP-bi-apt and Cell-Tracker-Deep-Red-labeled NK cells. After that, the cell mixture was subjected to flow cytometric analysis.

We then investigated whether the pre-binding of CP-bi-apt to CD16-positive NK cells would mask the effect of PD-L1 aptamer targeting. Briefly, 2×10^5 Cell-Tracker-Deep-Red-labeled NK cells were pre-treated with different aptamers (PD-L1-apt, CD16apt, CmutP-bi-apt, CPmut-bi-apt, or CP-bi-apt) at 37°C for 30 min. Then, the aptamer pre-treated NK cells were further co-incubated with 2×10^5 CFSE-labeled cancer cells at 37°C for 1 h. To verify the specificity of CP-bi-apt to PD-L1, CFSE-labeled tumor cells were firstly blocked

with anti-PD-L1 antibody and then co-incubated with CP-bi-apt pre-treated NK cells. After co-incubation, the cell mixtures were subjected to flow cytometric analysis.

CCK8 assay

NK cells, SMMC-7721, HeLa, and QSG-7701 cells (1×10^4) were seeded in 96-well plates, respectively. Twelve hours later, the cells were exposed to different doses of CP-bi-apt (0, 50, 100, 200, and 500 nM) at 37°C under a humidified atmosphere containing 5% CO_2. Twenty-four hours later, 10 μL CCK8 solution (Dojindo Laboratories, Kumamoto, Japan) and 90 μL DMEM medium were added to each well for another 2 h after discarding the original medium. The viability of cells was determined by the absorbance of formazan crystals through a plate reader (Spectra Max M5) at 450 nm. Cell viability was calculated using the following formula:

$$\text{Cell viability (\%)} = (OD_A - OD_{blank})/(OD_B - OD_{blank}) \times 100\%$$

Here, the OD_A and OD_B were the absorbance values of the treated and untreated cells, respectively. Moreover, OD_{blank} was the absorbance of CCK8 reagent itself. All experiments were performed in sextuplicate.

Luciferase-based lysis assay

To generate luciferase-expressing cell lines, HeLa cells and SMMC-7721 cells were infected with lentivirus encoding luciferase and were selected with puromycin. Then, HeLa-Luc and QSG-7701-luc cells (1×10^5) were seeded in 12-well plates, respectively. Twelve hours later, the cells were treated with NK cells (1×10^5) plus different aptamers (CD16-apt, PD-L1-apt, or CP-bi-apt; 200 nM) at 37°C for 3 h. Then, the original medium was discarded and washed with PBS. After that, 100 μL luciferase substrate was added after transferring the adherent cells into black 96-well plates. The bioluminescence of the above mixture was detected through a plate reader (Spectra Max M5) for evaluating the viability of tumor cells.

The apoptosis and cell death analysis

NK cells and target cells (tumor cells or non-cancerous cells) were mixed at the ratio of 1:1 and then incubated with 200 nM aptamer (CD16-apt, PD-L1-apt, or CP-bi-apt) at 37°C for 3 h. Then, the culture mediums were collected for determining granzyme B, perforin, TNF-α, and IFN-γ by using corresponding ELISA Kits (Boster Biological Technology, USA). Meanwhile, the cells were harvested and blocked off with 5% bovine serum albumin (BSA) (Sigma-Adrich, Germany) solution for 10 min and then stained with anti-CD16-APC antibody (eBioscience, USA) for an additional 30 min to distinguish NK cells. After washing with PBS, the cell mixture was collected and labeled with FITC-annexin V/ PI solution (Dojindo Laboratories) for 15 min. Finally, the proportion of apoptotic and necrotic cells to the total target cells was detected by flow cytometry. To better evaluate the function of CP-bi-apt, target cells without any treatment were regarded as the natural apoptosis group (control group).

Intracellular cytokine analysis by flow cytometry

To further evaluate the antitumor ability of NK cells assisted by CP-bi-apt, intracellular cytokines (granzyme B and perforin) of NK cells after different treatments were analyzed by flow cytometry. Briefly, 1×10^5 tumor cells were adhered in 12-well plates and then incubated with 1×10^5 NK cells and 200 nM of different aptamers (CD16-apt, PD-L1-apt, CP_{mut}-bi-apt, $C_{mut}P$-bi-apt, or CP-bi-apt; 200 nM) for 3 h. In this process, 1 μL/mL Golgi Stop TM Protein Transport Inhibitor (BD Pharmingen, USA) was added to block the intracellular transport processes. Subsequently, the cells were collected and washed with PBS and then fixed and permeabilized using the Cytofix/Cytoperm plus Fixation/Permeabilization kit (BD Pharmingen, USA). Then, the cells were harvested and blocked with 5% BSA solution for 10 min and stained with anti-CD56-FITC antibody (eBioscience, USA) for an additional 30 min to distinguish NK cells. After washing with PBS, the cell mixture was collected and labeled with anti-Perforin-APC (Cell Signaling Technology, USA) or anti-Granzyme B-PE (Cell Signaling Technology, USA), respectively. Finally, the stained cells were collected for further analysis by flow cytometry after washing with PBS.

In vivo biodistribution of CP-bi-apt and adoptive NK cells

Male BALB/c-nude mice were purchased from China Wushi (Shanghai, China). Ethical approval for animal studies was granted by Animal Ethics Committee of Mengchao Hepatobiliary Hospital of Fujian Medical University. Tumor-bearing mice were prepared by subcutaneously implanting SMMC-7721 tumor mass (the SMMC-7721 tumor model was prepared by subcutaneous injection with 1×10^7 SMMC-7721 cells) with the size about 1 mm^3. Until the tumor reached about 100 mm^3, the mice were randomly divided into four groups: PBS; CD16-apt; PD-L1-apt; and CP-bi-apt. These tumor-bearing mice were intravenously injected with PBS or equivalent Evagreen-labeled aptamers (0.7 nmol). At 24 h post-injection, the tumors and major organs were excised for fluorescence analysis by Gel Doc XR imaging system (Ex: 488 nm, 1 s).

Promoting the infiltration of NK cells into tumor tissues is essential for adoptive cell immunotherapy. Thus, SMMC-7721 tumor-bearing mice were also used to determine whether more NK cells were accumulated in tumor site with the assistance of CP-bi-apt. Briefly, this experiment divided into four groups, including PBS, NK cells, CD16-apt plus NK cells, PD-L1-apt plus NK cells, and CP-bi-apt plus NK cells. For visual tracking, the human NK cells were labeled with ICG-NHS before injection into mice. At 24 h post-injection, tumor and major organs (heart, liver, spleen, lung, and kidney) were excised for the analysis of ICG-labeled NK cells. All the fluorescence images were acquired under excitation at 808 nm with exposure of 300 ms. The fluorescence intensity was quantified by ImageJ.

In vivo antitumor efficacy of CP-bi-apt-based treatment

We further investigated the antitumor effect of CP-bi-apt and NK cells *in vivo* accordingly. Briefly, SMMC-7721 tumor-bearing mice were grouped (n = 5) and injected through tail vein every 2 days

with PBS, NK cells, CD16-apt plus NK cells, PD-L1-apt plus NK cells, CD16-apt plus PD-L1-apt plus NK cells, and CP-bi-apt plus NK cells, respectively. Each mouse received 5×10^6 NK cells, 1,000 units IL-2, and 0.7 nmol aptamers each time for a total of three injections. Tumor volumes were measured by digital caliper every second day and then calculated based on the following formula: tumor volume = (tumor length) $\times$ (tumor width)^2/2. Moreover, the mice were euthanized when the tumor reached a volume larger than 1,200 mm^3.

To further confirm the antitumor efficiency, the tumor-bearing mice (n = 3) underwent the aforementioned treatments. Three days after last injection, the tumors were surgically collected, fixed in 4% formalin solution, and embedded in paraffin for CD16, H&E, Ki67, PD-L1, and TUNEL (R&D Systems, USA) staining. For the assay of cytokines (granzyme B, perforin, IFN-γ, and TNF-α), part of the tumors in each group were weighed and homogenized with 1 mL PBS by automatic sample rapid grinding machine (Tissuelyser-24, JingXin, Shanghai). Subsequently, the supernatant was collected by centrifugation at 12,000 rpm for 10 min at 4°C and then evaluated by ELISA Kits (Boster Biological Technology, USA). All groups were experimented in triplicate.

Immunofluorescence and immunohistochemical staining

To visually show the intra-tumoral infiltration of NK cells, the paraffin sections of tumors in each group were dewaxed and hydrated. Antigen retrieval was performed using 10 mM sodium citrate buffer (pH = 9) at 95°C for 25 min in a Pascal pressure chamber (WanBaoPai, China). Then, the sections of tumors were performed by CD16 immunofluorescence staining (R&D Systems) and observed with confocal microscope. The fluorescence of DAPI was blue, while the fluorescence signal of CD16 was red. The intracellular cytokine (perforin and granzyme B) staining of NK cells has also been performed, and CD56 was used as the marker of NK cells for staining. For TUNEL assay, the corresponding tumor sections were incubated with TUNEL detecting buffer in dark for 2 h. The fluorescent images were recorded by confocal microscope. After antigen retrieval, the tumor sections were also stained with H&E, Ki67 antibody, or PD-L1 antibody for immunohistochemical analysis. The images were acquired with an optical microscope (Zeiss, Axiocam ERC5s) with a bright-field imaging system.

Biosafety analysis

To examine the biosafety of the aptamers *in vivo*, body weight of each mouse was recorded every second day after first treatment. Moreover, the fresh blood specimens of three mice in each group were collected and centrifuged to obtain serum for biochemical examination at 3 days post-treatment. The blood biochemical markers were analyzed with a biochemical autoanalyzer (CX5, Beckman Coulter Genomics, USA). The levels of ALP, ALT, AST, BUN, CREA, and GGT were examined to reflect the liver and kidney functions. Moreover, the possible toxicity was also evaluated by determining the morphological changes of the major organs (heart, liver, spleen, lung, and kidneys) in each group through H&E staining.

Statistical analysis

All results were presented as the mean ± SD. All statistical analyses were carried out using the Graphpad Prism 7.04 and performed using one-way ANOVA. Results with *$p < 0.05$, **$p < 0.01$, ***$p < 0.001$, and ****$p < 0.001$ were considered as statistically significant.

SUPPLEMENTAL INFORMATION

Supplemental information can be found online at https://doi.org/10.1016/j.omtn.2022.01.010.

ACKNOWLEDGMENTS

This work was supported by the National Natural Science Foundation of China (grant nos. 61727823 and 81871483), the Natural Science Foundation of Fujian Province (grant no. 2020J011157), and the Young and Middle-Aged Talents Training Project of Fujian Provincial Health Commission (grant no. 2019-ZQN-88).

AUTHOR CONTRIBUTIONS

A.Z., X.L., and J.L. designed and supervised the experiments. A.Z., Y.D., and Y.W. performed the experiments and analyzed the data. Y.Z., Z.N., M.W., C.Z., and D.Z. contributed to performing the experiments. A.Z. and Y.D. wrote the original draft. Writing, reviewing, and editing were executed by J.L. and X.L.

DECLARATION OF INTERESTS

The authors declare no competing interests.

REFERENCES

1. Hinrichs, C.S., and Rosenberg, S.A. (2014). Exploiting the curative potential of adoptive T-cell therapy for cancer. Immunol. Rev. *257*, 56–71.
2. Wang, W., Jiang, J., and Wu, C. (2020). CAR-NK for tumor immunotherapy: clinical transformation and future prospects. Cancer Lett. *472*, 175–180.
3. Rezvani, K., Rouce, R., Liu, E., and Shpall, E. (2017). Engineering natural killer cells for cancer immunotherapy. Mol. Ther. *25*, 1769–1781.
4. Bonifant, C.L., Jackson, H.J., Brentjens, R.J., and Curran, K.J. (2016). Toxicity and management in CAR T-cell therapy. Mol. Ther. Oncolytics. *3*, 16011.
5. Ingegnere, T., Mariotti, F.R., Pelosi, A., Quintarelli, C., De Angelis, B., Tumino, N., Besi, F., Cantoni, C., Locatelli, F., Vacca, P., et al. (2019). Human CAR NK cells: a new non-viral method allowing high efficient transfection and strong tumor cell killing. Front. Immunol. *10*, 957.
6. Davies, J.O.J., Stringaris, K., Barrett, A.J., and Rezvani, K. (2014). Opportunities and limitations of natural killer cells as adoptive therapy for malignant disease. Cytotherapy *16*, 1453–1466.
7. Childs, R.W., and Carlsten, M. (2015). Therapeutic approaches to enhance natural killer cell cytotoxicity against cancer: the force awakens. Nat. Rev. Drug Discov. *14*, 487–498.
8. Yang, S., Wen, J., Li, H., Xu, L., Liu, Y., Zhao, N., Zeng, Z., Qi, J., Jiang, W., Han, W., et al. (2019). Aptamer-engineered natural killer cells for cell-specific adaptive immunotherapy. Small *15*, e1900903.
9. Gasteiger, G., and Rudensky, A.Y. (2014). Interactions between innate and adaptive lymphocytes. Nat. Rev. Immunol. *14*, 631–639.
10. Bald, T., Krummel, M.F., Smyth, M.J., and Barry, K.C. (2020). The NK cell–cancer cycle: advances and new challenges in NK cell–based immunotherapies. Nat. Immunol. *21*, 835–847.
11. Siegler, E.L., Zhu, Y., Wang, P., and Yang, L. (2018). Off-the-Shelf CAR-NK cells for cancer immunotherapy. Cell Stem Cell *23*, 160–161.

12. Macdonald, J., Henri, J., Roy, K., Hays, E., Bauer, M., Veedu, R.N., Pouliot, N., and Shigdar, S. (2018). EpCAM immunotherapy versus specific targeted delivery of drugs. Cancers *10*, 19.

13. Carrega, P., Bonaccorsi, I., Di Carlo, E., Morandi, B., Paul, P., Rizzello, V., Cipollone, G., Navarra, G., Mingari, M.C., Moretta, L., et al. (2014). CD56 bright perforin low noncytotoxic human NK cells are abundant in both healthy and neoplastic solid tissues and recirculate to secondary lymphoid organs via afferent lymph. J. Immunol. *192*, 3805–3815.

14. Ku, T.H., Zhang, T., Luo, H., Yen, T.M., Chen, P.W., Han, Y., and Lo, Y.H. (2015). Nucleic acid aptamers: an emerging tool for biotechnology and biomedical sensing. Sensors *15*, 16281–16313.

15. Fang, X., and Tan, W. (2010). Aptamers generated from cell-SELEX for molecular medicine: a chemical biology approach. Acc. Chem. Res. *43*, 48–57.

16. Zhou, J., and Rossi, J. (2017). Aptamers as targeted therapeutics: current potential and challenges. Nat. Rev. Drug Discov. *16*, 181–202.

17. Morita, Y., Leslie, M., Kameyama, H., Volk, D., and Tanaka, T. (2018). Aptamer therapeutics in cancer: current and future. Cancers *10*, 80.

18. Hori, S.I., Herrera, A., Rossi, J.J., and Zhou, J. (2018). Current advances in aptamers for cancer diagnosis and therapy. Cancers *10*, 9.

19. Ohuchi, S. (2012). Cell-Selex technology. Biores. Open Access *1*, 265–272.

20. Zhou, G., Wilson, G., Hebbard, L., Duan, W., Liddle, C., George, J., and Qiao, L. (2016). Aptamers: a promising chemical antibody for cancer therapy. Oncotarget *7*, 13446–13463.

21. Bai, C., Gao, S., Hu, S., Liu, X., Li, H., Dong, J., Huang, A., Zhu, L., Zhou, P., Li, S., et al. (2020). Self-assembled multivalent aptamer nanoparticles with potential CAR-like characteristics could activate T cells and inhibit melanoma growth. Mol. Ther. Oncolytics *17*, 9–20.

22. Lao, Y.H., Phua, K.K.L., and Leong, K.W. (2015). Aptamer nanomedicine for cancer therapeutics: barriers and potential for translation. ACS Nano *9*, 2235–2254.

23. Shu, D., Zhang, L., Bai, X., Yu, J., and Guo, P. (2021). Stoichiometry of multi-specific immune checkpoint RNA Abs for T cell activation and tumor inhibition using ultra-stable RNA nanoparticles. Mol. Ther. Nucleic Acid *24*, 426–435.

24. Boltz, A., Piater, B., Toleikis, L., Guenther, R., Kolmar, H., and Hock, B. (2011). Bi-specific aptamers mediating tumor cell lysis. J. Biol. Chem. *286*, 21896–21905.

25. Tang, Z., Shangguan, D., Wang, K., Shi, H., Sefah, K., Mallikratchy, P., Chen, H.W., Li, Y., and Tan, W. (2007). Selection of aptamers for molecular recognition and characterization of cancer cells. Anal. Chem. *79*, 4900–4907.

26. McNamara, J.O., Kolonias, D., Pastor, F., Mittler, R.S., Chen, L., Giangrande, P.H., Sullenger, B., and Gilboa, E. (2008). Multivalent 4-1BB binding aptamers costimulate CD8+ T cells and inhibit tumor growth in mice. J. Clin. Invest. *118*, 376–386.

27. Li, Z., Hu, Y., An, Y., Duan, J., Li, X., and Yang, X.D. (2019). Novel bispecific aptamer enhances immune cytotoxicity against MUC1-positive tumor cells by MUC1-CD16 dual targeting. Molecules *24*, 478.

28. Soldevilla, M.M., Villanueva, H., Casares, N., Lasarte, J.J., Bendandi, M., Inoges, S., de Cerio, A.L.-D., and Pastor, F. (2016). MRP1-CD28 bi-specific oligonucleotide aptamers: target costimulation to drug-resistant melanoma cancer stem cells. Oncotarget *7*, 23182–23196.

29. Ganji, A., Varasteh, A., and Sankian, M. (2016). Aptamers: new arrows to target dendritic cells. J. Drug Target. *24*, 1–12.

30. Yang, Y., Sun, X., Xu, J., Cui, C., Yazd, H.S., Pan, X., Zhu, Y., Chen, X., Li, X., Li, J., et al. (2020). Circular bispecific aptamer-mediated artificial intercellular recognition for targeted T cell immunotherapy circular bispecific aptamer-mediated artificial intercellular recognition for targeted T cell immunotherapy. ACS Nano *14*, 9562–9571.

31. Vandghanooni, S., Eskandani, M., Barar, J., and Omidi, Y. (2018). Bispecific therapeutic aptamers for targeted therapy of cancer: a review on cellular perspective. J. Mol. Med. *96*, 885–902.

32. Benson, D.M., Bakan, C.E., Mishra, A., Hofmeister, C.C., Efebera, Y., Becknell, B., Baiocchi, R.A., Zhang, J., Yu, J., Smith, M.K., et al. (2010). The PD-1/PD-L1 axis modulates the natural killer cell versus multiple myeloma effect: a therapeutic target for CT-011, a novel monoclonal anti-PD-1 antibody. Blood *116*, 2286–2294.

33. Chen, L., and Han, X. (2015). Anti-PD-1/PD-L1 therapy of human cancer: past, present, and future. J. Clin. Invest. *125*, 3384–3391.

34. Prodeus, A., Abdul-Wahid, A., Fischer, N.W., Huang, E.H.B., Cydzik, M., and Gariépy, J. (2015). Targeting the PD-1/PD-L1 immune evasion Axis with DNA aptamers as a novel therapeutic strategy for the treatment of disseminated cancers. Mol. Ther. Nucleic Acids *4*, e237.

35. Yu, L., Hu, Y., Duan, J., and Yang, X.D. (2015). A novel approach of targeted immunotherapy against adenocarcinoma cells with nanoparticles modified by CD16 and MUC1 aptamers. J. Nanomater. *2015*, 316968. https://doi.org/10.1155/2015/316968.

36. Topalian, S.L., Drake, C.G., and Pardoll, D.M. (2012). Targeting the PD-1/B7-H1(PD-L1) pathway to activate anti-tumor immunity. Curr. Opin. Immunol. *24*, 207–212.

37. Di Giusto, D.A., Knox, S.M., Lai, Y., Tyrelle, G.D., Aung, M.T., and King, G.C. (2006). Multitasking by multivalent circularDNA aptamers. ChemBioChem *7*, 535–544.

38. Nishino, M., Ramaiya, N.H., Hatabu, H., and Hodi, F.S. (2017). Monitoring immune-checkpoint blockade: response evaluation and biomarker development. Nat. Rev. Clin. Oncol. *14*, 655–668.

39. Pardoll, D.M. (2012). The blockade of immune checkpoints in cancer immunotherapy. Nat. Rev. Cancer *12*, 252–264.

40. Lai, W.-Y., Huang, B.-T., Wang, J.-W., Lin, P.-Y., and Yang, P.-C. (2016). A novel PD-L1-targeting antagonistic DNA aptamer with antitumor effects. Mol. Ther. Nucleic Acids *5*, e397.

41. Zhang, D., Zheng, Y., Lin, Z., Liu, X., Li, J., Yang, H., and Tan, W. (2020). Equipping natural killer cells with specific targeting and checkpoint blocking aptamers for enhanced adoptive immunotherapy in solid tumors. Angew. Chem. *132*, 12120–12126.

42. Dong, W., Wu, X., Ma, S., Wang, Y., Nalin, A.P., Zhu, Z., Zhang, J., Benson, D.M., He, K., Caligiuri, M.A., et al. (2019). The mechanism of anti-PD-L1 antibody efficacy against PD-L1-negative tumors identifies NK cells expressing PD-L1 as a cytolytic effector. Cancer Discov. *9*, 1422–1437.

43. Poznanski, S.M., Ritchie, T.M., Fan, I.Y., El-Sayes, A., Portillo, A.L., Ben-Avi, R., Rojas, E.A., Chew, M.V., Shargall, Y., and Ashkar, A.A. (2021). Expanded human NK cells from lung cancer patients sensitize patients' PDL1-negative tumors to PD1-blockade therapy. J. Immunother. Cancer *9*, e001933.

OMTN, Volume *27*

Supplemental information

CD16/PD-L1 bi-specific aptamer for cancer immunotherapy through re-cruiting NK cells and acting as immunocheckpoint blockade

Aixian Zheng, Yanlin Du, Yiru Wang, Youshi Zheng, Zhaoyu Ning, Ming Wu, Cuilin Zhang, Da Zhang, Jingfeng Liu, and Xiaolong Liu

Table S1. The sequences of aptamers used in this study

Name	Detailed sequence
CD16-apt	5'-(P)-GACCTGCCCACTGCGGGGGTCTATACGTGAGGA<br>AGAAGTGGGCAGGTCCAGACGGGCTTTTTTTTTTTTT<br>TTTTTTTTTTTTTTTTTTTTGCCCGTCTG-3'
PD-L1-apt	5'-(P)-GACCTGCCCACTTTTTTTTTTTTTTTTTTTTTTT<br>AGTGGGCAGGTCCAGACGGGCCACATCAACTCATTGAT<br>AGACAATGCGTCCACTGCCCGTCTG-3'
CPmut-bi-apt	5'-(P)-GACCTGCCCACTGCGGGGGTCTATACGTGAGGA<br>AGAAGTGGGCAGGTCCAGACGGGCCACATCAACTCAT<br>T***C***ATA***C***ACAAT***C***C***C***TCCACTGCCCGTCTG-3'
CmutP-bi-apt	5'-(P)-GACCTGCCCACT***C***CGG***C***GGT***G***TATAC***C***TGAG***C***AA<br>GAAGTGGGCAGGTCCAGACGGGCCACATCAACTCATT<br>GATAGACAATGCGTCCACTGCCCGTCTG-3'
CP-bi-apt	5'-(P)-GACCTGCCCACTGCGGGGGTCTATACGTGAGGA<br>AGAAGTGGGCAGGTCCAGACGGGCCACATCAACTCAT<br>TGATAGACAATGCGTCCACTGCCCGTCTG-3'

The letters with straight underline and wavy underline represent the sequences of CD16 aptamer and PD-L1 aptamer, respectively. CD16-apt and PD-L1-apt were constructed by only replacing the bases of counter aptamer in the loop of the CP-bi-apt with T bases. CmutP-bi-apt and CP_{mut}-bi-apt are the aptamers with some mutations, which were marked in bold italics. All of them are used as control.

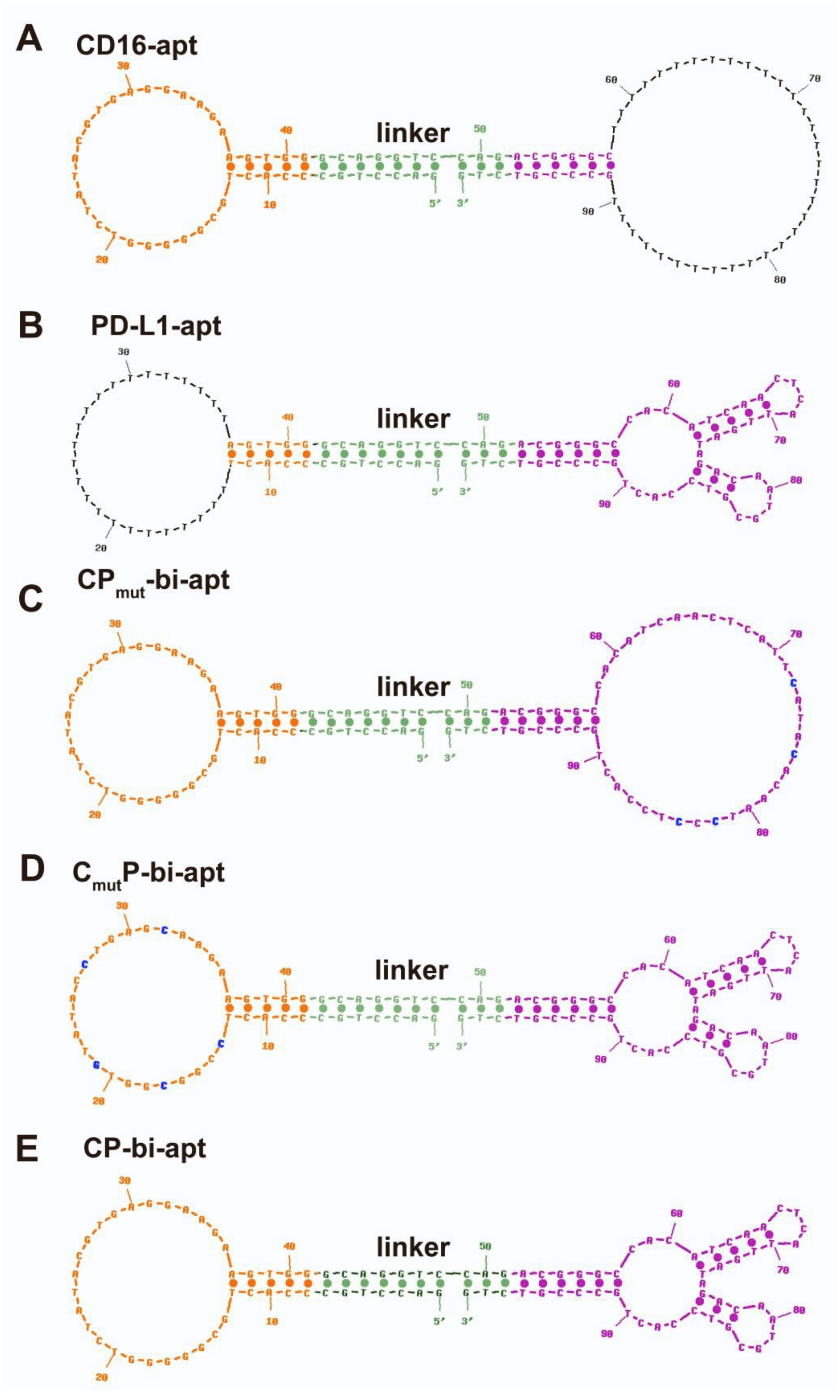

Figure S1. The predicted structures of the aptamers used in this study. The letters in green represent the sequences of linker.

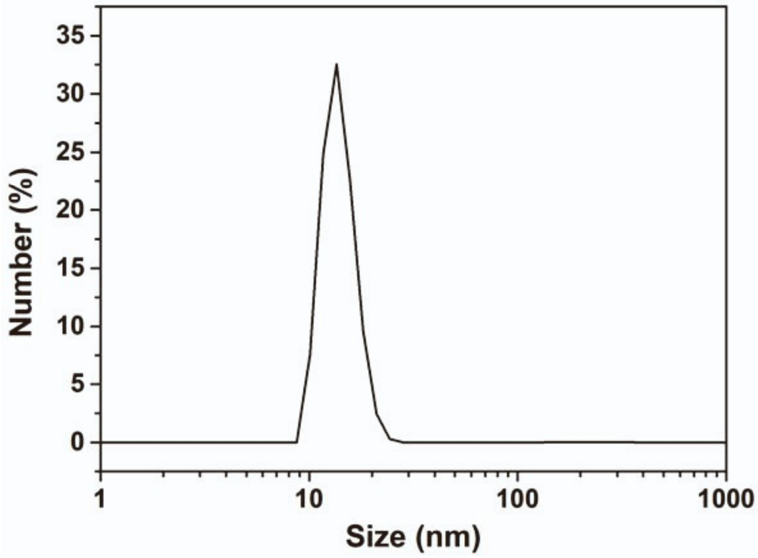

Figure S2. The hydrodynamic size of CP-bi-apt measured by Dynamic Light Scattering (DLS).

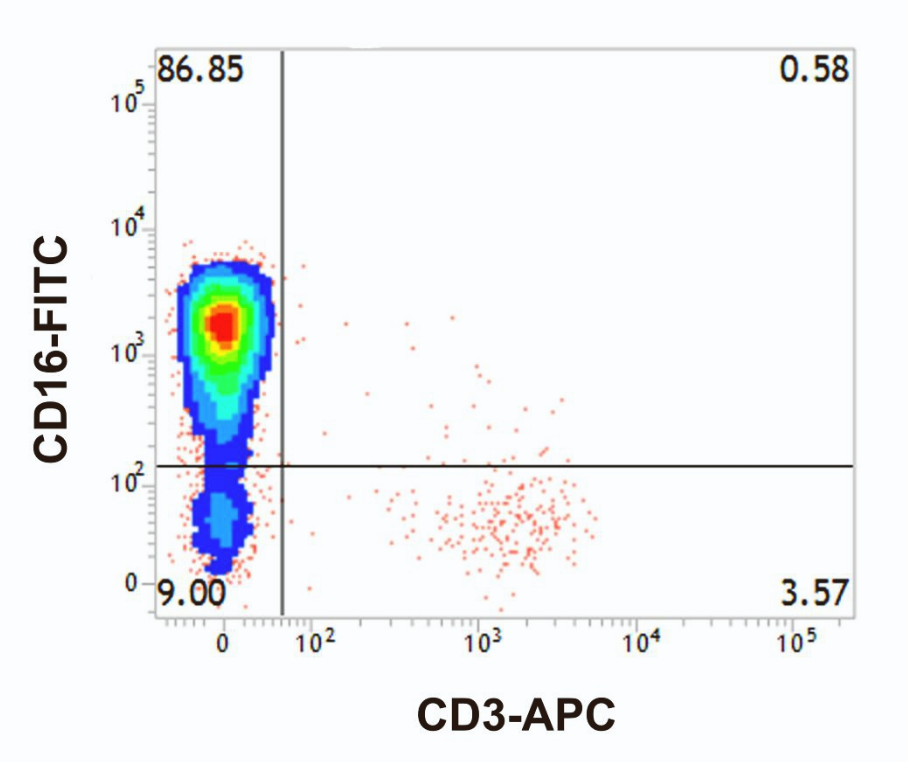

Figure S3. The expression of CD16 in human NK cells. NK cells were stained with anti-CD3-APC and anti-CD16-FITC before determining by flow cytometry.

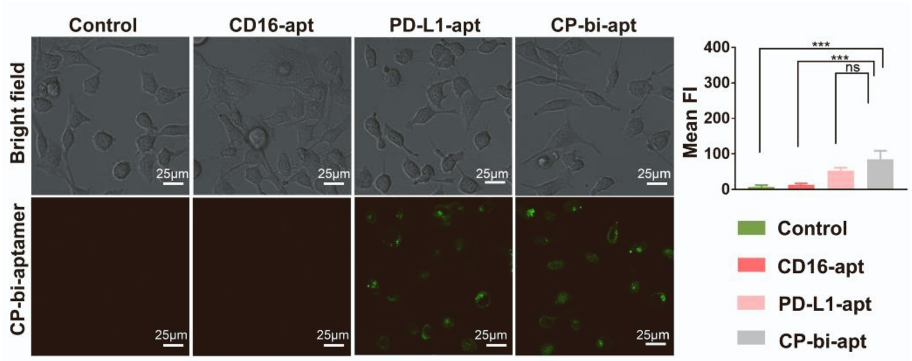

Figure S4. The binding ability of CP-bi-apt to QSG-7701 cells. QSG-7701 cells were incubated with 200 nM Evagreen-labeled different aptamers (CD16-apt, PD-L1-apt and CP-bi-apt) for 30 min. The fluorescence images were detected by confocal microscope (488 nm).

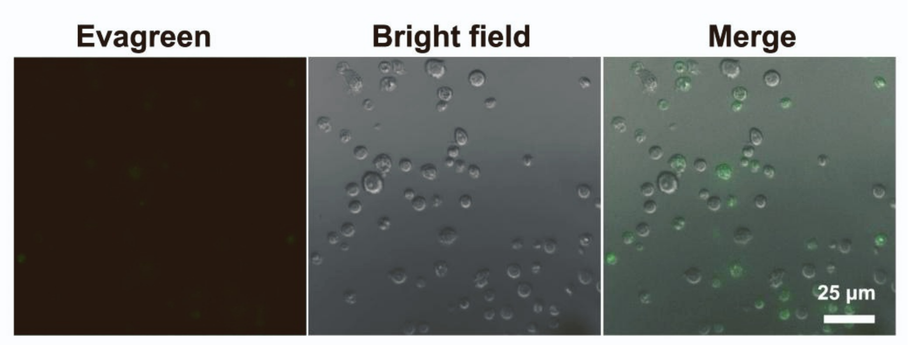

Figure S5. Confocal fluorescence images of murine NK cells incubated with Evagreen labeled CP-bi-apt (200 nM).

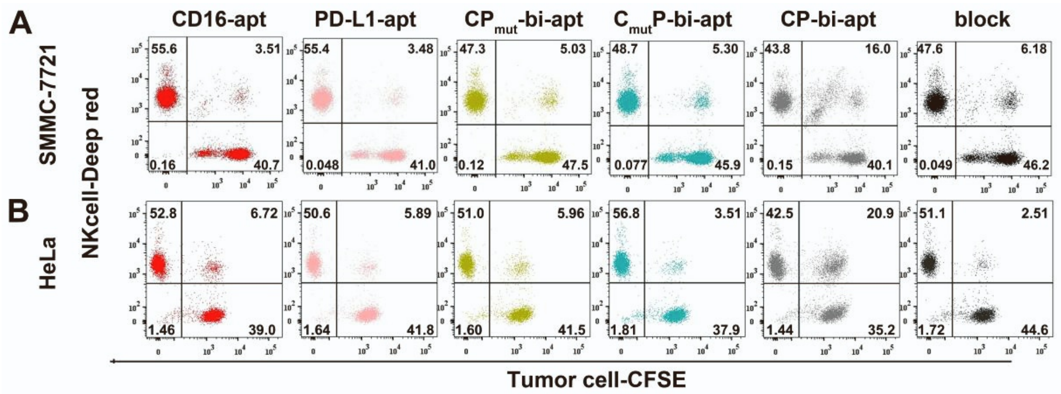

Figure S6. Flow cytometric analysis of CFSE-labeled (A) SMMC-7721 cells or (B) HeLa cells after co-incubating with Cell-Tracker Deep Red-labeled NK cells at 37℃ for 60 min. NK cells were pretreated with different aptamers (PD-L1-apt, CD16-apt, C_{mut}P-bi-apt, CP$_{mut}$-bi-apt or CP-bi-apt) at 37℃ for 30 min.

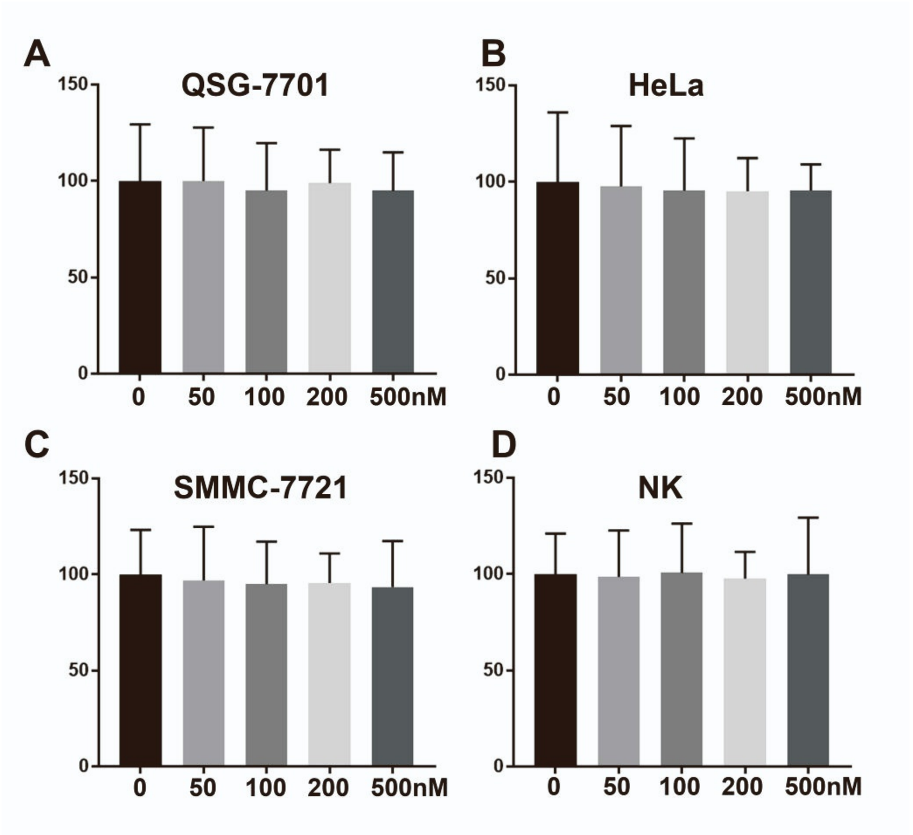

Figure S7. The in vitro toxicity of CP-bi-apt in different cells analyzed by CCK8 assay. Cell viability of (A) QSG-7701, (B) HeLa, (C) SMMC-7721 and (D) NK cells after co-incubation with different concentrations of CP-bi-apt (0, 50, 100, 200, 500 nM) for 24 h.

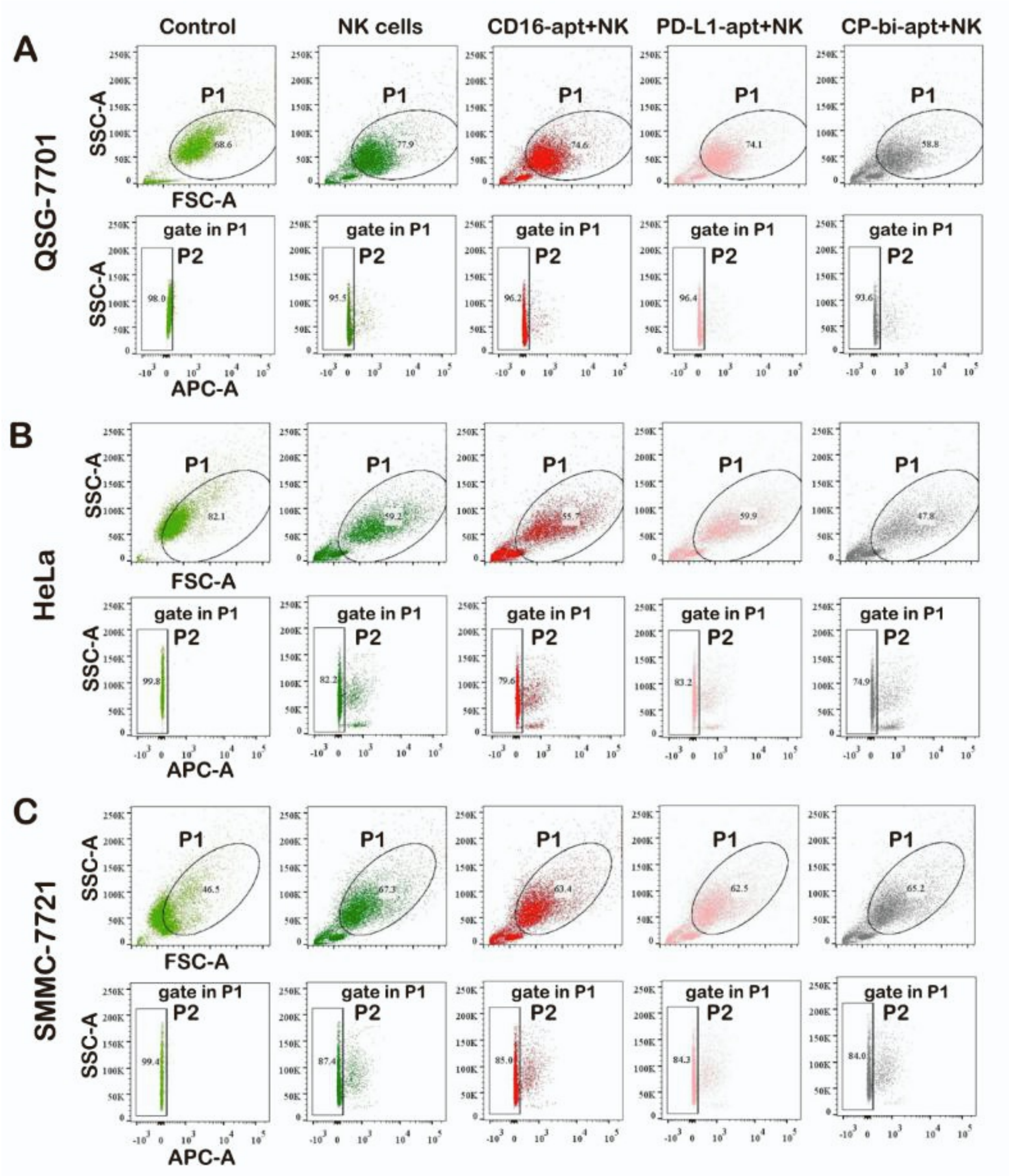

Figure S8. The gating strategy for flow cytometry analysis of (A) QSG-7701, (B) HeLa and (C) SMMC-7721 cells in the presence of NK cells and different aptamers (PD-L1-apt, CD16-apt or CP-bi-apt).

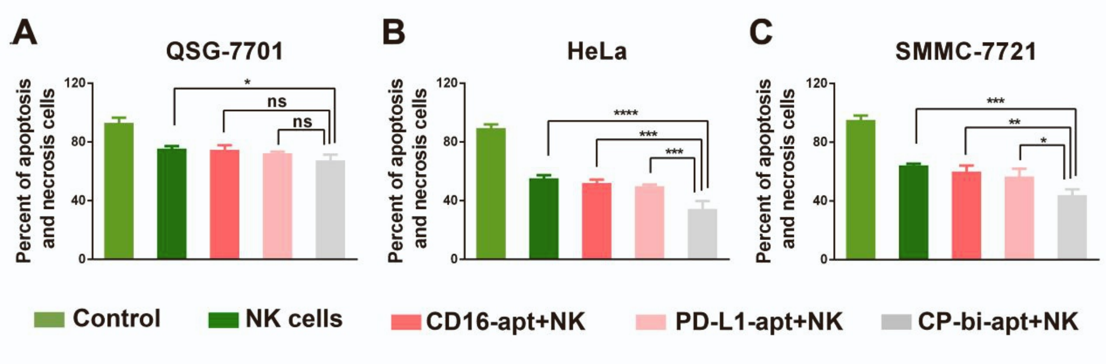

Figure S9. Flow cytometry analysis for evaluating the killing effect. Percentage of apoptosis and necrosis of (A) QSG-7701, (B) HeLa and (C) SMMC-7721cells after incubation with NK cells and different aptamers. The data represents the average result of three independent experiments.

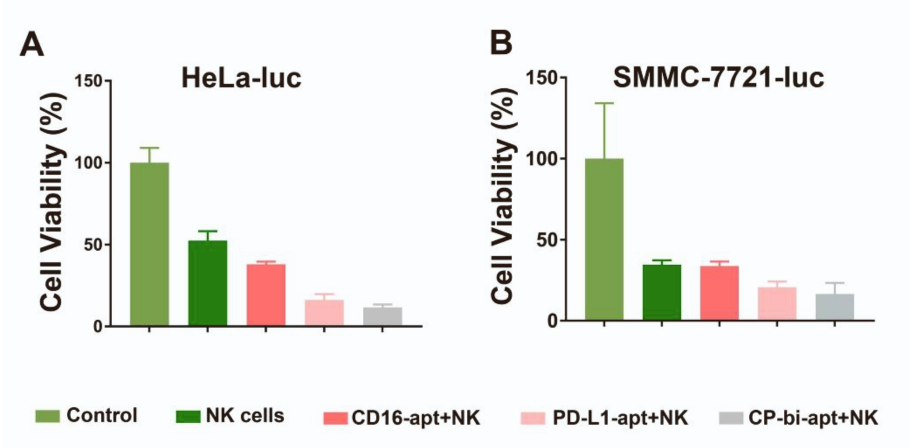

Figure S10. The luciferase based lysis assay for evaluating the killing effect. Cell viability of (A) HeLa-luc and (B) SMMC-7721-luc after co-incubation with NK cells and different kinds of aptamers (PD-L1-apt, CD16-apt or CP-bi-apt) for 3h.

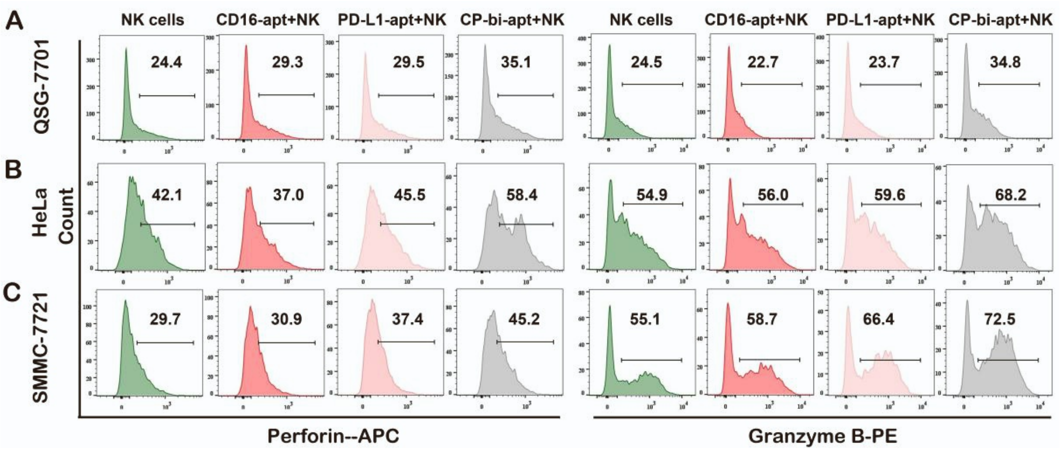

Figure S11. Flow cytometric analysis of intracellular Granzyme B and perforin of NK cells after co-incubating with different aptamers and (D) QSG-7701, (E) HeLa or (F) SMMC-7721 cells. The above experiments are all gated on CD56(+) cells by using FITC-conjugated anti-CD56 antibody.

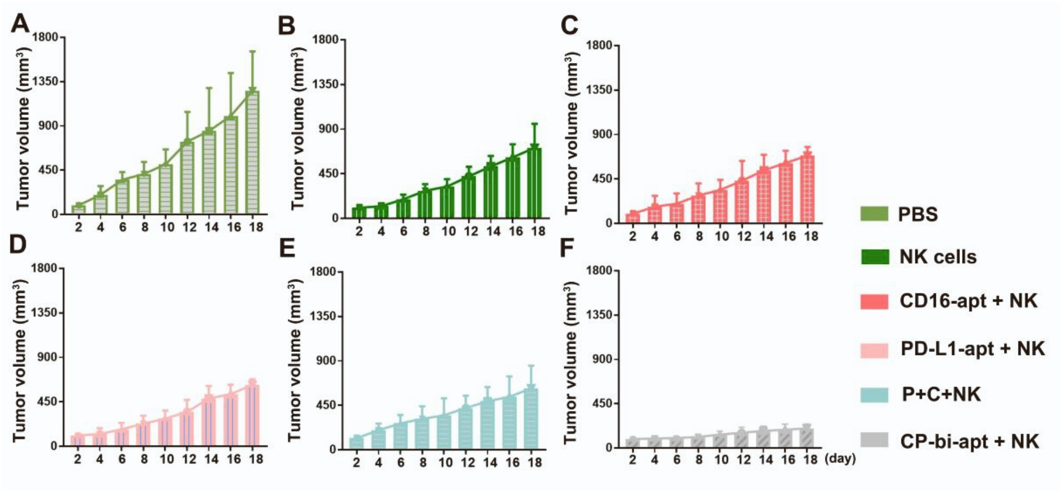

Figure S12. The individual growth curves of the tumors after various treatments (PBS, free NK cells, CD16-apt + NK cells, PD-L1-apt + NK cells, CD16-apt + PD-L1-apt +NK cells, CP-bi-apt + NK cells, n=5).

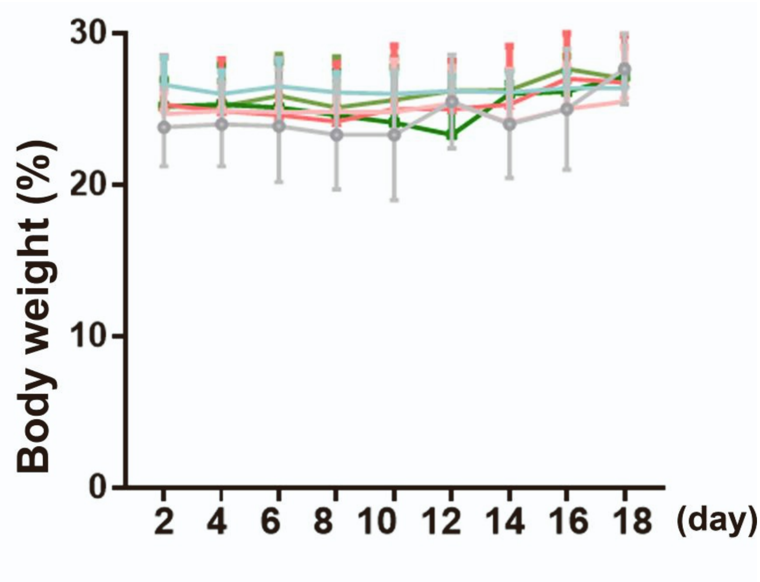

Figure S13. The body weights of the mice after various treatments (PBS, free NK cells, CD16-apt + NK cells, PD-L1-apt + NK cells, CD16-apt + PD-L1-apt +NK cells, CP-bi-apt + NK cells, n=5).

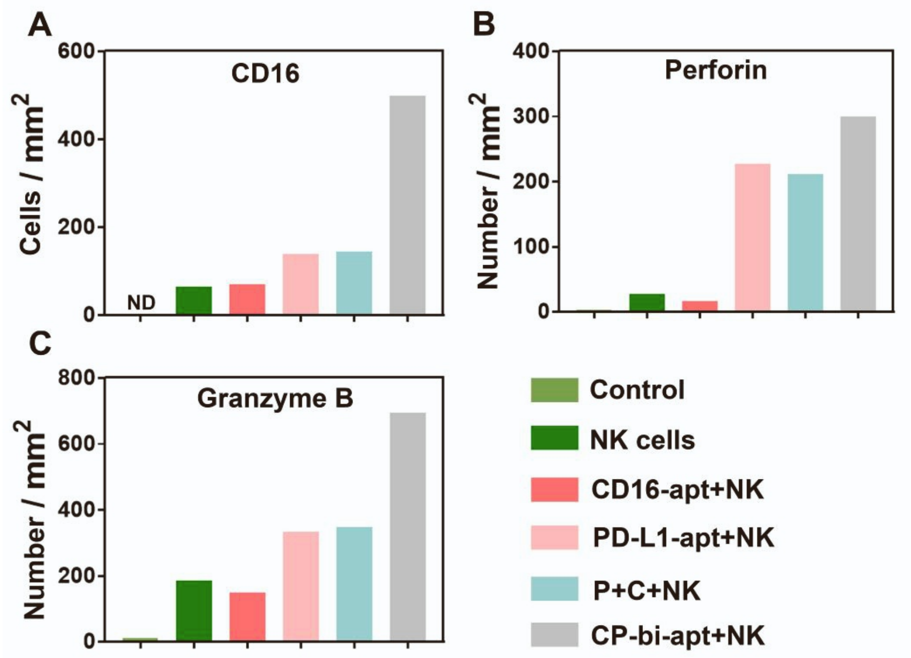

Figure S14. The statistical results of (A) CD16, (B) perforin and (C) Granzyme B immunofluorescence staining shown in Figure 6.

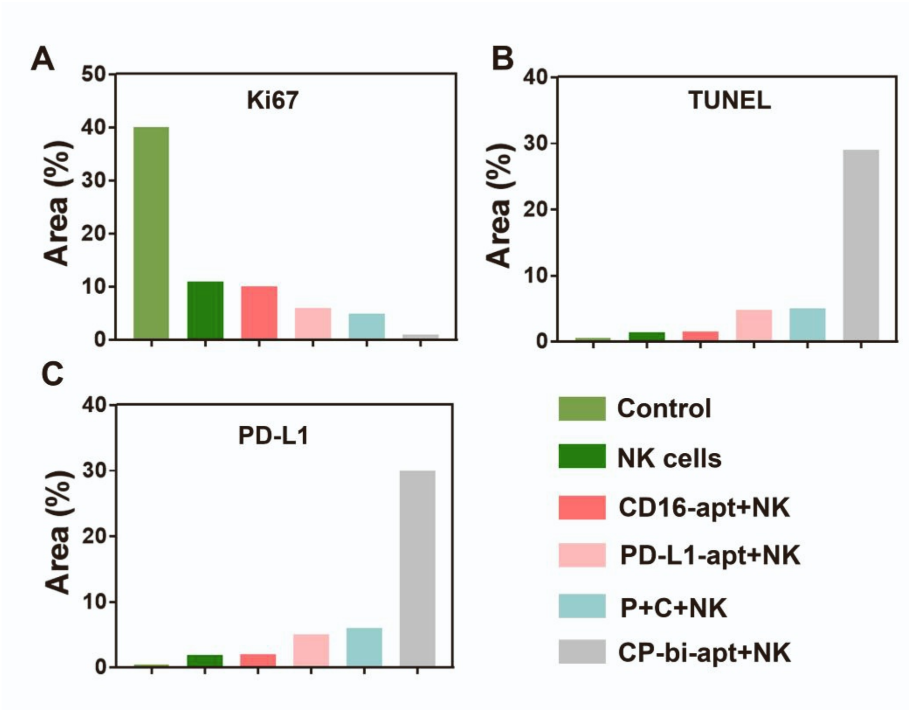

Figure S15. The statistical results of (A) Ki67, (B) TUNEL and (C) PD-L1 staining shown in Figure 7.

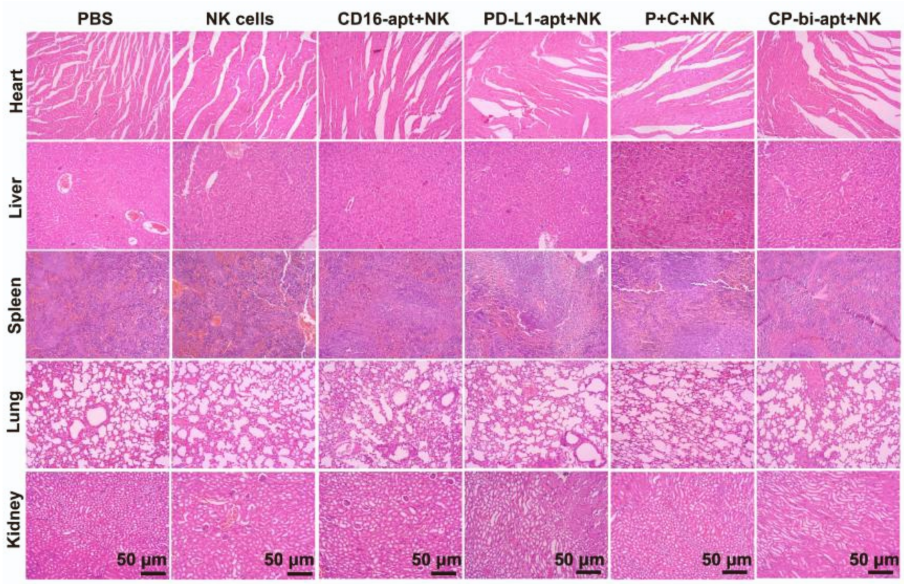

Figure S16. Histological images of the major organs (heart, liver, spleen, lung, and kidney) of mice with indicated treatments after the staining with H&E. Scale bar: 50 μm.

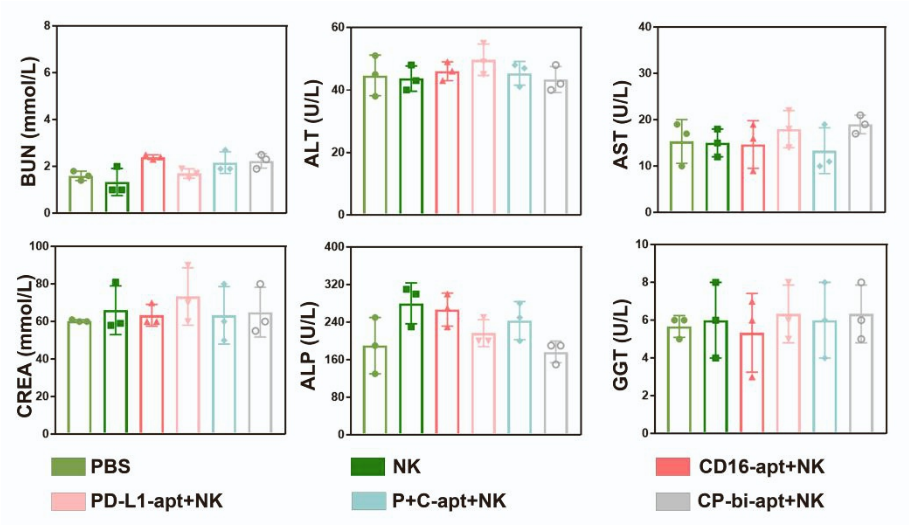

Figure S17. The analysis of serum biochemical indexes of the SMMC-7721 tumor-bearing mice with indicated treatments at three days after last injection. n = 3.
